# Discrimination of pancreato-biliary cancer and pancreatitis patients by non-invasive liquid biopsy

**DOI:** 10.1186/s12943-024-01943-x

**Published:** 2024-02-02

**Authors:** Christina Hartwig, Jan Müller, Hagen Klett, Dina Kouhestani, Anke Mittelstädt, Anna Anthuber, Paul David, Maximilian Brunner, Anne Jacobsen, Karolina Glanz, Izabela Swierzy, Lotta Roßdeutsch, Bettina Klösch, Robert Grützmann, Timo Wittenberger, Kai Sohn, Georg F. Weber

**Affiliations:** 1 Innovation Field In-vitro Diagnostics, Fraunhofer Institute for Interfacial Engineering and Biotechnology IGB, Stuttgart, Germany; 2 Institute for Interfacial Engineering and Plasma Technology IGVP, University of Stuttgart, Stuttgart, Germany; 3 Center for Integrative Bioinformatics Vienna (CIBIV), Max Perutz Labs, University of Vienna and Medical University of Vienna, Vienna BioCenter, Vienna, Austria; 4 Vienna BioCenter PhD Program, Doctoral School of the University of Vienna and Medical University of Vienna, Vienna, Austria; 5 Genedata GmbH, Munich, Germany; 6 Department of Surgery, Friedrich-Alexander University (FAU) Erlangen-Nürnberg, Universitätsklinikum Erlangen, Erlangen, Germany; 7 Deutsches Zentrum für Immuntherapie, Friedrich-Alexander-Universität Erlangen-Nürnberg, Universitätsklinikum Erlangen, Erlangen, Germany; 8 Comprehensive Cancer Center, Friedrich-Alexander-Universität Erlangen-Nürnberg, Universitätsklinikum Erlangen, Erlangen, Germany; 9 Bavarian Cancer Research Center (BZKF), Erlangen, Germany

**Keywords:** cfDNA, Next-generation sequencing, Pancreato-biliary cancer, Pancreatitis, Non-invasive diagnostics, Hybridization and capture, Methylation, cfMBD-Seq, DMRs, IPMN

## Abstract

**Background:**

Current diagnostics for the detection of pancreato-biliary cancers (PBCs) need to be optimized. We therefore propose that methylated cell-free DNA (cfDNA) derived from non-invasive liquid biopsies serves as a novel biomarker with the ability to discriminate pancreato-biliary cancers from non-cancer pancreatitis patients.

**Methods:**

Differentially methylated regions (DMRs) from plasma cfDNA between PBCs, pancreatitis and clinical control samples conditions were identified by next-generation sequencing after enrichment using methyl-binding domains and database searches to generate a discriminatory panel for a hybridization and capture assay with subsequent targeted high throughput sequencing.

**Results:**

The hybridization and capture panel, covering around 74 kb in total, was applied to sequence a cohort of 25 PBCs, 25 pancreatitis patients, 25 clinical controls, and seven cases of Intraductal Papillary Mucinous Neoplasia (IPMN). An unbiased machine learning approach identified the 50 most discriminatory methylation markers for the discrimination of PBC from pancreatitis and controls resulting in an AUROC of 0.85 and 0.88 for a training (*n* = 45) and a validation (*n* = 37) data set, respectively. The panel was also able to distinguish high grade from low grade IPMN samples.

**Conclusions:**

We present a proof of concept for a methylation biomarker panel with better performance and improved discriminatory power than the current clinical marker CA19-9 for the discrimination of pancreato-biliary cancers from non-cancerous pancreatitis patients and clinical controls. This workflow might be used in future diagnostics for the detection of precancerous lesions, e.g. the identification of high grade IPMNs vs. low grade IPMNs.

**Supplementary Information:**

The online version contains supplementary material available at 10.1186/s12943-024-01943-x.

Pancreatic cancer is the eleventh most common cancer type and the seventh leading cause of cancer deaths [1]. The 5-year survival rate is only up to 12% [2], however, at present there is no diagnostic biomarker available with sufficient diagnostic power for early and reliable diagnostics [3]. Pancreatic ductal adenocarcinoma (PDAC) is most often detected as secondary finding or when unspecific symptoms appear which typically occur in advanced tumor stages [4]. Additionally, those symptoms (e.g. jaundice) can be similar for various cancers including distal bile duct, pancreatic (head), and ampullary cancers. If local treatment is possible, pancreaticoduodendectomy (Whipple procedure) is the surgical treatment of choice. This indicates the reasonability to aim for a combined diagnostic approach for those pancreato-biliary cancers. The discrimination between pancreatic cancer and other pancreatic diseases such as pancreatitis, known as a risk factor for malignant transformation and pancreatic cancer itself, can be challenging [5].

To this end, a robust biomarker with good accessibility (e.g. non-invasive), specificity and sensitivity is a prerequisite to discriminate between cancers of pancreato-biliary origin (hereafter PBC), including bile duct, pancreatic, and ampullary cancers as well as non-malignant diseases. Such a biomarker could help to facilitate clinical decision-making regarding adequate surgery or therapy (e.g. radiation, chemotherapy or endoscopic cholangiopancreatography). A promising class of biomarker candidates for highly sensitive and specific diagnostic approaches represents circulating cell-free DNA (cfDNA) which can be isolated from blood plasma. In this context, analyzing epigenetic modifications including methylation of cfDNA reveals “cancer-specific signatures” [6, 7]. Consequently, we aimed to specifically enrich methylated cfDNA using a methyl-binding domain with subsequent high-throughput sequencing (cfMBD-Seq) from plasma of PBC as well as pancreatitis patients in this proof of concept study (Fig. 1A). Based on these analyses, we identified differentially methylated regions (DMRs) as targets with potential discrimination power of different pancreatic diseases. Finally, we validated our target regions by the establishment of a corresponding hybridization and capture approach as a robust and more economical procedure for clinical translation (Fig. 1B). More information about the used materials and methods can be found in the Supplementary Methods (Additional File 1).

**Fig. 1:**
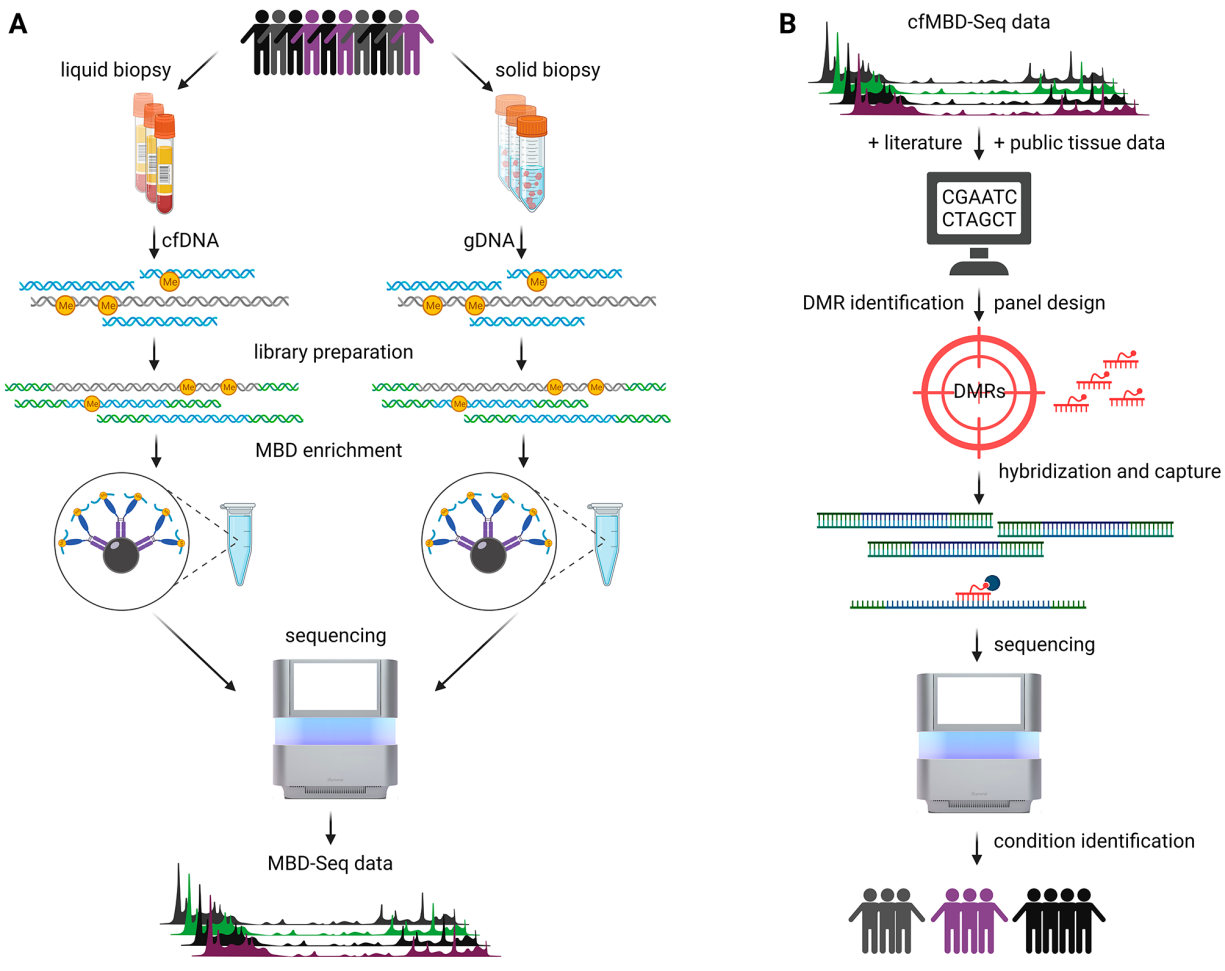
General workflow. **A**: DNA was isolated of liquid and solid biopsies derived from different patient cohorts. Sequencing libraries were prepared that underwent methyl-binding domain (MBD) enrichment. The enriched fragments were sequenced by means of NGS to generate MBD-Seq data. **B**: CfMBD-Seq data together with already published regions from literature and public tissue data were used to identify differentially methylated regions (DMRs) that served for the design of a targeted panel. The panel was used for hybridization and capture with subsequent sequencing to enable high-throughput identification of different patient subgroups

## Results and discussion

We developed a workflow for the discrimination of PBCs from non-cancerous pancreatitis samples and clinical controls, by analyzing the epigenetic landscape of cell-free DNA in human patient plasma samples of 115 individuals in total that were included in our study (Supplementary Table 1; Additional File 2 and Supplementary Fig. 1; Additional File 3). Most PBC patients of this study were at stage II (41%) and stage III (38%), while less patients were at early stages (3% stage 0 and 9% stage I) and stage IV (9%). Additional information about clinical characteristics, neoadjuvant therapy, staging, grading, and resection margin of PBC patients are summarized in Supplementary Tables 2 and 3 (Additional File 4 and 5). A characterization of pancreatitis patients (Supplementary Table 4; Additional File 6) and IPMN patients (Supplementary Table 5; Additional File 7) are also provided.

Although CA19-9, a well-established marker for pancreatic cancers, is routinely used in the clinics, the median was below the clinical threshold of 37 U/ml for the non-PDAC PBC subgroup (Supplementary Table 1; Additional File 2). To define target regions for a diagnostic panel based on hybrid capture sequencing to discriminate between clinically relevant conditions including pancreato-biliary cancer (PBC), pancreatitis, and controls, empirical cfMBD-Seq data were generated from 11 PDAC patients, 6 non-PDAC cancer patients, 8 pancreatitis patients, 4 clinical controls, and 12 healthy controls for diagnostic DMR selection (from cohort C1, see Supplementary Fig. 1; Additional File 3). PDAC and non-PDAC cohorts were considered as one group (PBC) because of similarity in surgical treatment and/or symptomatology. Sequencing data displayed a non-random distribution across the genome and an accumulation of methylated cfDNA fragments in distinct regions including CpG islands and promoters (Supplementary Fig. 2A; Additional File 8). In a next step, we identified differentially methylated regions (DMRs) between samples from PBC (pancreatic ductal adenocarcinoma (PDAC) combined with non-PDAC) and pancreatitis as well as from controls and healthy individuals that were merged for each group, respectively (Supplementary Fig. 2B; Additional File 8). Additionally, data of sorted cancer cells from seven corresponding patients (EpCAM^+^ cells of PBC patients) were also inspected for corresponding cancer specific signals. DMRs ranked under the identified top 120 showed already promising performance for discrimination of PBCs, pancreatitis and healthy (Supplementary Fig. 3; Additional File 9).

In this context, the three subgroups that make up the PBC group showed no subtype-specific grouping by PCA analysis (Supplementary Fig. 4; Additional File 10), indicating similar methylation patterns between PDAC and non-PDAC cancer samples. In addition, already published regions from two reviews (Henriksen and Thorlacius-Ussing, 2021 [8] and Al Shaheri et al., 2021 [9]) were selected to complement the target panel. Moreover, tissue DMRs identified from public pancreatic tumor and normal tissue samples (TCGA, GSE49149) also contributed to the selection of regions specific for PBC. Finally, the panel comprised 233 DMRs covering roughly 74 kb in total which were used for hybrid capture enrichment (see Supplementary Table 6; Additional File 11 and Supplementary Fig. 5; Additional File 12).

Using our panel for hybrid capture enrichment, we sequenced 15 PBC, 15 controls, and 15 pancreatitis. Sequencing data of this identification cohort C2 (see Supplementary Fig. 1, Additional File 3) were used to confirm the diagnostic potential of the identified putative biomarkers for discrimination of PBC, pancreatitis, and controls. To this end, samples were selected to exclude gender, age, and body-mass-index as confounding factors (Supplementary Fig. 6; Additional File 13). The hybrid capture enrichment panel comprised 9544 quality-filtered single CpGs, of which the most informative and discriminatory 50 CpGs were identified using an unbiased machine learning approach (see next paragraph) to further optimize the discriminatory power (Supplementary Table 7; Additional file 14). 60.0% of these 50 most discriminatory DMCs (30/50) were found in promoters or their direct proximity (distances to promoter: 29/30 < = 1 kb, 1/30 1.6 kb), 22.0% (11/50) in distal intergenic regions, 14.0% (7/50) in introns, and 4.0% (2/50) of DMCs in exons. Remarkably, 12 of the identified DMCs were found to have a direct link to cancer. For example, eight DMCs were found in the promoter region of the APC regulator of WNT signaling pathway (APC) which is already known to be mutated in most human colorectal cancers. Another two DMCs with potential link to pancreatic cancer were found in the promoter region of COL4A1, which is a gene for an extracellular matrix protein. It belongs to the collagen type IV proteins that have been described to contribute to the perineural invasion of pancreatic cancer cells [10].

**Fig. 2:**
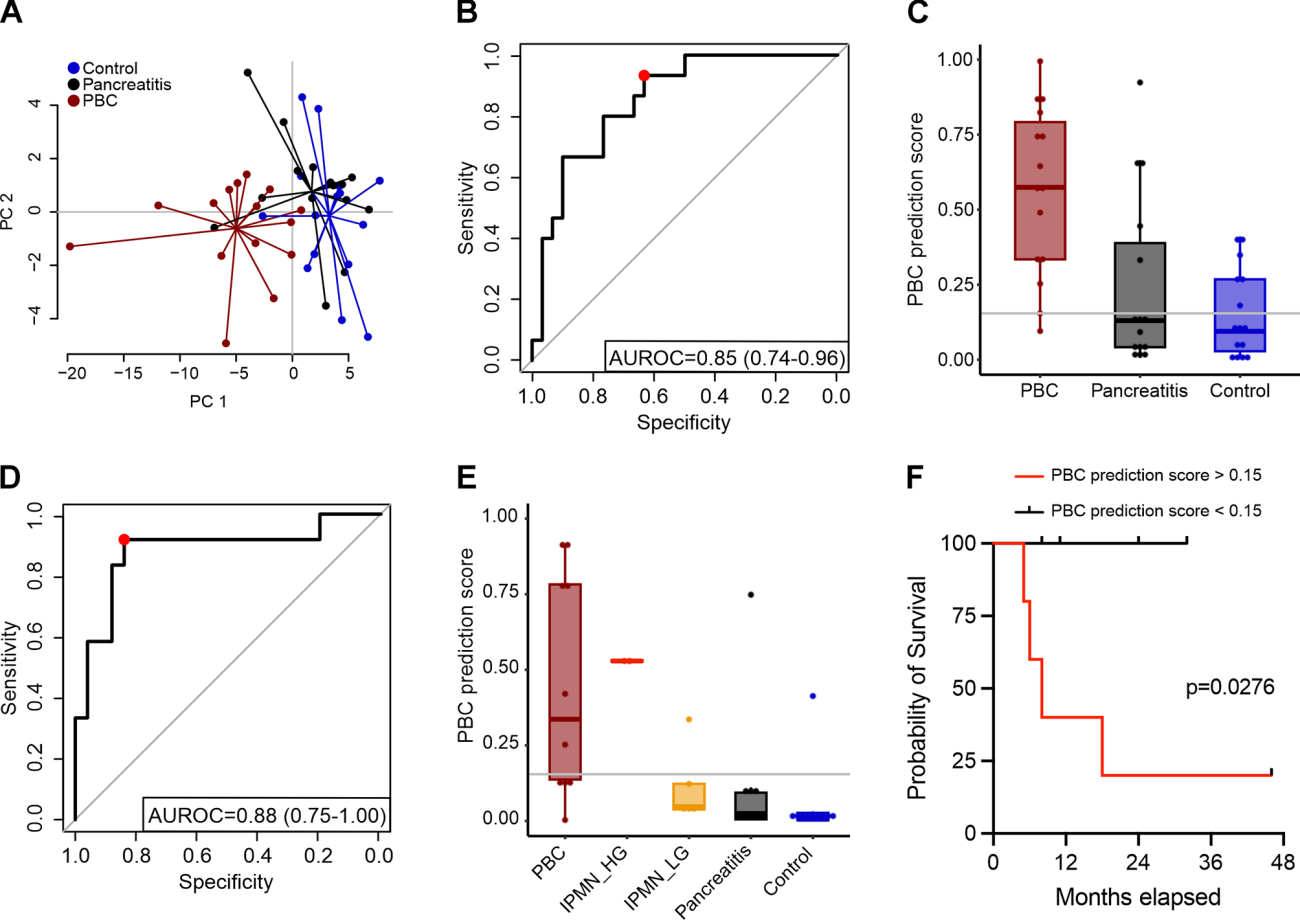
Machine learning approach M1. 50 most promising DMCs (hybridization and capture approach) combined with CA19-9 values for distinguishing PBC from pancreatitis and controls. **A**: PCA based on the 50 most informative DMCs combined with CA19-9 values for the conditions control (blue), pancreatitis (black), and PBC (red). Variances explained: PC1 = 56.75%, PC2 = 9.05%. **B**: ROC curve (AUC = 0.85) of PBC predicition scores for the identification cohort C2. The red dot indicates the determined optimal threshold value for the PBC prediction score that maximizes sensitivity and specificity with a defined minimum sensitivity of 90%. **C**: Boxplot of PBC prediction scores from the identification cohort C2 with the optimized classification threshold of 0.15 (gray line). **D**: ROC curve (AUC = 0.88) of PBC prediction scores for the validation cohort C3 including IMPNs. The red dot indicates the threshold value for classifying PBCs and high grade IPMNs with a minimum sensitivity of 90%. **E**: Boxplot of the PBC prediction scores from the validation cohort C3 including low and high grade IPMNs and the pre-determined PBC classification threshold of 0.15 (gray line). **F**: Kaplan-Meier curve for the survival of PBC patients from the validation cohort C3. Follow-up of 44 months after diagnosis. Separation of PBC group (*n* = 10) into two subgroups by the pre-determined PBC classification threshold of 0.15

To evaluate the predictive power of liquid biopsy data from our targeted panel, we used an unbiased machine learning approach to discriminate cancers of pancreato-biliary origin (PBCs) from patients with pancreatitis and clinical controls. The data was split into an identification cohort C2 for feature selection and model training (Supplementary Fig. 7; Additional File 15) and a validation cohort C3 for model performance evaluation. The best performing machine learning model (M1) also included CA19-9 concentrations. The PCA of M1 already showed a good separation of PBC samples from pancreatitis and control samples (Fig. 2A) without any subtype-specific separation of the three subgroups that make up the PBC group (Supplementary Fig. 8; Additional File 16). Cross-validation on the identification cohort C2 of M1 (samples from 15 PBCs, 15 pancreatitis patients and 15 clinical controls) resulted in overall AUROC = 0.85, Sensitivity = 0.93 and Specificity = 0.63 based on a feature set of 50 methylation sites and CA19-9 concentrations (Fig. 2B). An optimal classification of PBC samples was achieved using a threshold of 0.15 for the SVM prediction score (Fig. 2C and Supplementary Fig. 9A; Additional file 17). To validate the performance of the machine learning approach M1 we analyzed an independent validation cohort C3 (Supplementary Fig. 1; Additional File 3, Supplementary Table 8; Additional File 18, and Supplementary Fig. 10; Additional File 19) consisting of 10 PBCs, 10 pancreatitis patients, and 10 clinical controls as well as 7 IPMNs (2 high grade and 5 low grade) to also test for discrimination of high vs. low grade IPMNs and to potentially identify early stages of cancer. This independent validation cohort showed an overall performance of AUROC = 0.88, Sensitivity = 0.92 and Specificity = 0.84 in detecting positive/‘intention to treat’ samples, meaning PBCs and high grade IPMNs (Fig. 2D and E). Studies using cfDNA methylation comparing pancreatic cancer patients in all stages solely with healthy controls (but not for discrimination from pancratitis) revealed sensitivities of 81%, 93%, and 97% with specificities of 85%, 89%, and 92%, respectively [11–13]. As target regions from these publications were also considered for our panel design, we are convinced that our hybridization and capture panel comprises the most informative regions that were known to date. Interestingly, of the reported DNA-methylation signatures for the differentiation of pancreatic cancer from chronic pancreatitis by Wu Y et al. [14] only one methylation site is included in our presented panel (cg15138289 (HLA-DPB1, gene body)). The reason for this might be due to different cohorts, underlying methods and approaches to identify marker regions. Remarkably, high grade IPMNs could be also distinguished from low grade IPMNs in our test set as well (Supplementary Fig. 9B; Additional file 17). These results imply that our targeted panel has potential to discriminate PBCs and high grade IPMNs from low grade IPMNs, pancreatitis patients, and clinical controls.

Strikingly, single DMCs from our analyses performed significantly better than the tumor marker CA19-9 alone (AUROC = 0.720) with AUROC scores of 0.878 and 0.892 for two exemplary individual CpGs in the identification cohort C2 (One-sided DeLong’s test: *p* = 0.012 and *p* = 0.009, Supplementary Fig. 11; Additional File 20 and Supplementary Fig. 12; Additional File 21). Of note, our results suggest that the selected methylation markers of our targeted panel increase the prediction performance compared to the clinical standard of care tumor marker CA19-9.

Finally, we also evaluated the prognostic potential of our classification model. The validation set included 10 PBCs with a median follow up of 19.1 months (Fig. 2F). All detected (correctly classified) PBCs have a significantly shorter survival time (median survival of 8 months) as compared to the non-detected PBCs (*p* = 0.0276). Interestingly, patients with PBCs with a short overall survival (follow up of 44 months after diagnosis) have been accurately detected in the validation set (Fig. 2F and Supplementary Fig. 9; Additional File 17). The machine learning approach did not detect all PBCs, but there might be a bias towards specifically detecting more aggressive PBCs that should be investigated in larger cohorts.

Similar to another study to solely discriminate PDAC from pancreatitis with promising markers (e.g. protein kinase C beta type; accuracy 100%) [14] our study also suffers from a relatively small cohort size where more comprehensive patient groups and a multi-center setting would be desirable, of course. In this context, some clinical cases of interest like pancreatitis and IPMN are comparably rare and could not be recruited on a larger scale within the scope of this study.

## Conclusions

Taken together, we demonstrate proof of concept for a hybridization and capture method to reliably identify pancreato-biliary cancer patients. The designed panel enables diagnostics in the same organ by discriminating pancreato-biliary cancers from pancreatitis with better performance than current standard of care. The presented workflow entails potential for clinical application and could possibly be used for cost-effective screening of e.g. risk groups [15]. Our work is paving the way for future studies to improve non-invasive diagnostics for reliable and potentially earlier detection of pancreato-biliary cancers. Moreover, applying this concept to other cancer types and clinical indications could help to improve diagnostics on a larger scale.

### Electronic supplementary material

Below is the link to the electronic supplementary material.

**Additional File 1**: Supplementary Information

**Additional File 2**: Baseline characteristics of the study cohort

**Additional File 3** Overview of 115 individual patients included in the two phases of the study

**Additional File 4** Tumor characteristics of patients with PBC (*n* = 40)

**Additional File 5**: Tumor characteristics of patients with PBC according to subtype (I – III)

**Additional File 6**: Characteristics of pancreatitis patients

**Additional File 7**: Characteristics of patients with Intraductal Papillary Mucinous Neoplasia (IPMN)

**Additional File 8**: Visualization of cfMBD-Seq data

**Additional File 9**: Analysis of cfMBD-Seq data for detection of DMRs by methylaction

**Additional File 10**: PCA of cfMBD-Seq data with DMRs identified by methylaction

**Additional File 11**: 233 DMRs covered by the hybridization and capture panel

**Additional File 12**: Venn diagram for the 233 DMRs covered by the hybridization and capture sequencing panel

**Additional File 13**: Validation of cohort characteristics of cohort C2

**Additional File 14**: 50 DMCs identified as putative biomarkers by the hybridization and capture panel

**Additional File 15**: Schematic view of the machine learning approach for unbiased performance evaluation

**Additional File 16**: PCA based on the putative biomarkers identified with the hybridization and capture approach

**Additional File 17**: Machine learning approach M1

**Additional File 18**: Comparison of identification cohort C2 with validation cohort C3 of the machine learning approach

**Additional File 19**: Beta values of identification cohort C2 and validation cohort C3

**Additional File 20**: Methylation markers on different chromosomes identified with the hybridization and capture sequencing

**Additional File 21**: Machine learning approach CA19-9

**Additional File 22**: TRIPOD Checklist

**Additional File 23**: TCGA-GDC sample information

**Additional File 24**: Graphical abstract

## Data Availability

The raw datasets generated for the current study are freely available in the Sequencing Read Archive (SRA) of NCBI under the accession number PRJNA1005578 (https://www.ncbi.nlm.nih.gov/bioproject/PRJNA1005578).

## Abbreviations

cell: free DNA (cfDNA)
cell: free DNA methyl-binding domain sequencing (cfMBD-Seq)
CI: confidence interval
ctDNA: circulating tumor DNA
CT: computed tomography
DMC: differentially methylated CpG
DMR: differentially methylated region
IPMN: intraductal papillary mucinous neoplasia
MRI: magnetic resonance imaging
non-PDAC: non-pancreatic ductal adenocarcinoma
PBC: pancreato-biliary cancer
PCA: principal component analysis
PDAC: pancreatic ductal adenocarcinoma
