# Supplementary material for: Discrimination of pancreato-biliary cancer and pancreatitis patients by non-invasive liquid biopsy
Source: Mol Cancer. 2024 Feb 2;23:28. doi: 10.1186/s12943-024-01943-x (PMC10836044; doi:10.1186/s12943-024-01943-x)
Supplement: Supplementary file 1 — Additional File 1: Supplementary Information [file 12943_2024_1943_MOESM1_ESM.docx]

**Additional File 1, Supplementary Information**

**Discrimination of pancreato-biliary cancer and pancreatitis patients by non-invasive liquid biopsy**

Christina Hartwig^1,2^, Jan Müller^1,3,4^, Hagen Klett^5^, Dina Kouhestani^6^, Anke Mittelstädt^6^, Anna Anthuber^6^, Paul David^6^, Maximilian Brunner^6^, Anne Jacobsen^6^, Karolina Glanz^1^, Izabela Swierzy^6^, Lotta Roßdeutsch^6^, Bettina Klösch^6^, Robert Grützmann^6,7,8,9^, Timo Wittenberger^5^, Kai Sohn^1*^, Georg F. Weber^6,7,8,9*^

1 Innovation Field In-vitro Diagnostics, Fraunhofer Institute for Interfacial Engineering and Biotechnology IGB, Stuttgart, Germany

2 Institute for Interfacial Engineering and Plasma Technology IGVP, University of Stuttgart, Stuttgart, Germany

3 Center for Integrative Bioinformatics Vienna (CIBIV), Max Perutz Labs, University of Vienna and Medical University of Vienna, Vienna BioCenter, Vienna, Austria

4 Vienna BioCenter PhD Program, Doctoral School of the University of Vienna and Medical University of Vienna, Vienna, Austria

5 Genedata GmbH, Munich, Germany

6 Department of Surgery, Friedrich-Alexander University (FAU) Erlangen-Nürnberg, Universitätsklinikum Erlangen, Erlangen, Germany

7 Deutsches Zentrum für Immuntherapie, Friedrich-Alexander-Universität Erlangen-Nürnberg, Universitätsklinikum Erlangen, Erlangen, Germany

8 Comprehensive Cancer Center (CCC) Erlangen-EMN, Friedrich-Alexander University (FAU) Erlangen-Nürnberg and Universitätsklinikum Erlangen, Erlangen, Germany.

9 Bavarian Cancer Research Center (BZKF), Erlangen, Germany

* Shared senior authorship and corresponding authors: Kai Sohn (Kai.Sohn@igb.fraunhofer.de) and Georg F. Weber (Georg.Weber@uk-erlangen.de)

**Supplementary Methods**

**Patient cohort:** Patients over 18 years were recruited at the University Hospital of Erlangen with the approval of the local ethics committee and the clinical trial number 180_19 B between 2019 and 2023. Clinical follow-up was conducted until 2023 (48 months). Plasma samples have been collected before surgery. Blood/DNA samples of 40 patients with histologically proven pancreato-biliary adenocarcinoma (PBC), 7 intraductal papillary mucinous neoplasm (IPMN), 30 chronic pancreatitis, and 26 patients that underwent clinical surgery for non-malignant diseases (clinical controls) were used in this study as well as blood plasma of 12 healthy volunteers acquired commercially from Biomex GmbH (Supplementary Table 1, Additional File 2 and Supplementary Table 2, Additional File 4). PDAC and non-PDAC cancer patients were summarized in the pancreato-biliary cancer (PBC) group with the three subgroups I (PDAC in the head of the pancreas), II (PDAC in the body and tail of the pancreas), and III (non-PDAC including acinic cell carcinoma, distal bile duct/biliary cancer, duodenal cancer, and ampullary/papillary cancer; Supplementary Table 3, Additional File 5). All experiments were performed in accordance with the study protocol approved by the ethics committee. Written informed consent was provided by all patients included into the study.

Additionally, we followed the reporting guidelines of TRIPOD and attached the corresponding TRIPOD checklist for a transparent documentation of the study (see Additional File 22).

**EpCAM+ sorted cells and tumor tissue DNA:** Fresh tissue collected during surgery from PBC and pancreatitis patients were minced into small enzymatically dissociated single cells using 3 ml of digestion media (DMEM, 2% FCS, 1 mg/ml collagenase XI) for 30 min at 37 °C. For the preparation of single cell suspension, digested cells were filtered using a 40 µm filter and washed twice with 10 ml of wash medium (DMEM, 2% FCS, 0.1% BSA). Sorting of EpCAM^+^ cells from the single cell suspension was performed using MoFlo Astrios EQ1 (Beckmann Coulter, Indianapolis, IN 46268, United States). Anti-EpCAM PE-CF594 (Biolegend, Clone-9C4, catalog no -324231) and anti-CD45 Pacific blue (Biolegend, clone - HI30, catalog no – 304022) antibodies were used to separate tumor cells (EpCAM^+^) from hematopoietic cells (CD45^+^) and fibroblast (EpCAM^-^CD45^-^). EpCAM^+^ cells were resuspended in 100 µl of ATL buffer. Tumor tissue DNA (TTDNA) was isolated using QIAamp DNA Micro Kit (50) according to the manufacturer’s recommendation.

**Plasma preparation and cfDNA isolation:** Human peripheral blood was centrifuged at 350 ×g for 10 min without brake (acceleration 9, deaccelaration 4) at room temperature. Preparation of blood plasma and cfDNA isolation was performed similar to an already published workflow [16, 17]: Plasma was centrifuged for 10 min at 16,000 ×g and 4 °C. CfDNA isolation was performed in an automated manner using a QIAsymphony® SP DNA Preparation System with the QIAsymphony DSP Circulating DNA Kit according to the instruction manual. 1.1-4 ml plasma served as input. Alternatively, cfDNA was isolated by means of the QIAcube with the QIAamp MinElute ccfDNA Mini Kit according to the manufacturer’s instructions.

**Cell-free Methyl-CpG-Binding Domain sequencing:** 3 ng of each isolated cfDNA sample was barcoded by using the adapter ligation-based NEXTflex Cell Free DNA-Seq Kit 2.0, until the ligation step with 1:30 diluted adapters and an additional heat inactivation of the ligase for 5 min at 65 °C. Afterwards, 90 µl of each barcoded sample were pooled and purified with the given sample-to-bead ratio of 1:1 and eluted in 68 µl nuclease-free water. 65 µl of the eluate were used as input for methyl-CpG-binding domain enrichment with the EpiMark Methylated DNA Enrichment Kit. The protocol was performed according to the instruction manual until the elution step where the sample was eluted with 26 µl of nuclease-free water in a thermomixer set at 65 °C for 15 min at 1200 rpm. 23 µl of the eluate were used for final PCR of the NEXTflex sample prep protocol that was completed according to the manufacturer’s instructions. Final libraries consisted of a pool of barcoded, methylated cfDNA samples that were sequenced using a HiSeq 2500 (Illumina) or NextSeq 2000 (Illumina), generating 25-30 million single-end reads per sample.

**Hybridization and capture:** Methylation enrichment with the hybridization and capture method was performed using the NEBNext Enzymatic Methyl-seq Library Preparation Protocol (NEB) followed by target enrichment and sequencing according to the Twist Targeted Methylation Sequencing Protocol. The regions covered by the sequencing panel are shown in Supplementary Table 6, Additional File 11 and Supplementary Figure 5, Additional File 12. Final enriched libraries were sequenced on a NextSeq 2000 (Illumina) with a sequencing depth of 12.5 million paired-end read pairs (100 bp) corresponding to 25 million reads per sample.

**Quality control of DNA and libraries:** All samples were measured and quality-controlled by means of Qubit dsDNA HS Assay Kit (Thermo Fisher Scientific) and by the High Sensitivity NGS Fragment Kit (1-6000 bp) using Fragment Analyzer (Agilent) or Ultra-sensitivity NGS Analysis Kit using Femto Pulse (Agilent).

**Cell-free Methyl-CpG-Binding Domain sequencing data processing:** After initial quality control of raw sequencing reads with FastQC (v0.11.8), the following steps were performed [18]: 1. Removal of sequencing adapters, removal of terminal polyG sequences (min 10 sequential G’s), removal of reads shorter than 50 base pairs, and quality trimming (BBTools - bbduk.sh v38.67) [19]. 2. Processed reads were mapped to the human reference genome assembly GRCh37 using NextGenMap (v0.5.5) with default settings [20]. 3. Mapped reads were deduplicated with samtools rmdup (v1.9), and reads in blacklisted regions were removed with bedtools intersect (v2.30.0) [21–23]. Mapped reads were converted to BigWig format with deeptools (bin size = 10, normalization = counts per million (CPM), bamCoverage v3.5.1) for visualization in the Integrative Genomics Viewer (IGV, v2.11.9) [24, 25].

**Identification of target regions for the panel:** cfMBD-Seq data of 6 non-PDAC patients, 8 pancreatitis patients, 11 PDAC patients, and 12 healthy controls was used for DMR identification.

Methylaction and MEDIPS (sliding-window-based tools) were used to identify DMRs [26, 27]. DMRs on sex chromosomes were excluded to minimize potential erroneous signals arising from gender differences. Loci with low read coverage were excluded from the differential analysis (methylaction: mean read count of all samples per window ≥ 1, MEDIPS: sum of read counts of all samples per window ≥ 20). For methylaction, loci were considered as DMRs if the adjusted p-value was lower than 0.01 and if the log_2_ fold change was higher than 0.4. Additionally, DMRs were classified as uniquely methylated or unmethylated in each condition. For each type of uniquely methylated or unmethylated DMRs, DMRs were ranked by adjusted p value and selected the top 20, for a total of top 120 DMRs. For MEDIPS, loci were considered as DMRs if the adjusted p-value was lower than 0.1. For the sequencing panel, the top 120 DMRs from methylaction and the DMRs called by methylaction (regardless of rank) and MEDIPS concurrently were selected.

In addition to this sliding window approach, a peak calling based approach was applied to determine DMRs independently. Raw sequencing reads were quality controlled using FastQC (v0.11.9). Subsequently, Illumina universal adapters as well as low quality bases (< phred 20) were removed and a minimum length of 20 bp was obtained. Next, reads were aligned to the genome assembly GRCh38 using BWA MEM (v0.7.17). Furthermore, QSEA [28] was utilized to obtain quality metrics, such as non-CpG/CpG coverage fractions and duplicates were removed. Peaks of methylated regions were identified using SICER by comparing enriched with input control samples and were considered with a p-value < 1e-10 (SICER parameters: fragment size of 400 bp, 100 bp sliding window, and 100 bp gap). We consolidated peaks to common regions, requiring presence in at least 50% of samples per analysis group and normalized peak count. In a post-processing step, we focused on regions enriched with CpGs only (>= 20) and calculated DMRs based on log-transformed count data using moderated t-tests between PBC and healthy + pancreatitis samples. 43 regions were identified with a p-value cutoff < 1e-4 and effect size > 0.3 and considered for the targeted panel.

Previously published cfDNA methylation markers to distinguish PDAC, pancreatitis, and healthy samples were also added to the sequencing panel after visual inspection and manual curation based on signal strength and signal-to-noise ratio in our sequencing data.

In order to achieve better transferability to diagnostic applications with liquid biopsies, we decided to identify target regions primarily from liquid biopsy samples and to complement these only with target regions from tissue-based data.

**Public tissue data DMR selection:** Preprocessed data (beta values) from Illumina 450k DNA methylation arrays were downloaded for primary pancreatic cancer (TCGA n=184; GSE49149 n=155) and normal pancreatic samples (TCGA n=10; GSE49149 n=19). Subsequently, DMRs between tumor and normal samples were called using bumphunting, independently for TCGA and GSE49149. Next, regions were consolidated and quantified by calculating the arithmetic mean of CpGs per region to generate a data matrix (regions x samples). Target regions for the targeted panel were identified using two approaches: a) calculate delta beta values between tumor and normal samples and choose common regions among the top 20 and bottom 10 regions that were present in both the TCGA and GSE49149 study; b) use a support vector machine (SVM) to classify tumor vs normal and select top 10 DMRs with the best SVM ranking (predictive capability). In total, 23 unique DMRs (approach a) n=14; approach b) n=9) were identified and considered for the targeted panel.

**Targeted methylation sequencing data processing:** After initial quality control of raw sequencing reads with FastQC (v0.11.8), the following steps were performed [18]: 1. Removal of sequencing adapters, removal of terminal polyG sequences (min 10 sequential G’s), removal of reads shorter than 50 base pairs, and quality trimming (BBTools - bbduk.sh v38.67)[19]. 2. Processed reads were mapped to the human reference genome assembly GRCh37 using Bismark (v0.23.1) with bowtie2 and default settings [29]. 3. Mapped reads were deduplicated with Picard MarkDuplicates (v2.27.4), and reads with a mapping quality smaller than 2 were removed with samtools (v1.9)[21–23]. 4. The methylation states of CpGs were determined with Bismark, considering only CpGs in the predefined 233 target regions (Additional File 11).

**Data preprocessing for machine learning:** The following steps were taken to prepare the data for the machine learning approach. A) Preprocessed targeted methylation sequencing data was filtered for loci with sufficient coverage > 20 in all samples. B) Age dependent loci were identified (FDR < 0.2) and removed by fitting a linear regression model using the DSS package (v2.42.0) resulting in a total of 9478 CpG beta values.

**Machine learning biomarker identification by feature selection and model training:** The identification cohort was split into training and test sets using a 10-fold balanced cross validation (CV) split. Next, for each split model predictors - continuous beta values of CpGs - were ranked in the training set differentiating the positive (PBCs) and the control (pancreatitis and clinical controls) group using Wald’s test in the DSS package. The number of CpGs was optimized by comparing CV prediction results on the test sets using the top N = {5, 10, 15, 20, 30, 40, 50, 75, 100, 150} ranked methylation sites and log(CA19-9 + 1) transformed expression values [U/ml] resulting in N=50. No missing data was present. Subsequently, the top 50 CpGs from the average rank of methylation sites across the 10-fold CV and CA19-9 expression were chosen as final feature set. Furthermore, the cutoff for the PBC prediction score (SVM probabilities; Figure 2B and C) was optimized by maximizing the sum of sensitivity and specificity in the CV resulting in a cutoff = 0.15 (Ties in the sum of sensitivity and specificity were resolved by choosing the better sensitivity). Finally, a linear SVM classification model was trained using the entire identification cohort with the identified final feature set. The SVM classification model is available on demand as “svm” class of the R caret package.

**Validation:** To evaluate the performance of the classification model an untouched validation set (n=37) was predicted using the trained SVM classification model and the final feature set (no missing data). SVM probability predictions were translated into positive (PBC prediction score >= 0.15) and negative (PBC prediction score < 0.15) outcomes. Performance metrics were obtained by comparing prediction results (positive vs. negative) to observed outcomes (positive = PBCs + high-grade IPMNs; negative = pancreatitis + clinical controls + low-grade IPMNs).
