# Supplementary material for: Discrimination of pancreato-biliary cancer and pancreatitis patients by non-invasive liquid biopsy
Source: Mol Cancer. 2024 Feb 2;23:28. doi: 10.1186/s12943-024-01943-x (PMC10836044; doi:10.1186/s12943-024-01943-x)
Supplement: Supplementary file 2 — Additional File 2: Baseline characteristics of the study cohort [file 12943_2024_1943_MOESM2_ESM.docx]

|  | **All patients** | **Subgroups** | | | | | | |
| --- | --- | --- | --- | --- | --- | --- | --- | --- |
|  |  | **PBC** | | **IPMN** | **Pancreatitis** | **Controls** | **Healthy** | **p** |
|  |  | **PDAC** | **Non-PDAC** |  |  |  |  |  |
| **N (%)** | 115 (100) | 28 (24) | 12 (10) | 7 (6) | 30 (26) | 26 (23) | 12 (10) | - |
| **Age (years), median (IQR)** | 63 (22) | 71 (14) | 74 (17) | 73 (14) | 60 (14) | 61 (18) | 45 (7) | **< 0.001** |
| **Gender, n (%)**  **Female**  **Male** | 43 (37)  72 (63) | 13 (46)  15 (54) | 3 (25)  9 (75) | 3 (43)  4 (57) | 10 (33)  20 (67) | 11 (42)  15 (58) | 3 (25)  9 (75) | 0.695 |
| **BMI (kg/m^2^) (n=97)*, median (IQR)** | 24.3 (6.3) | 22.7 (6.4) | 26.3 (7.6) | 24.7 (7.0) | 22.8 (6.3) | 24.3 (5.8) | - | 0.517 |
| **ASA (n=93)*, n (%)**  **I**  **II**  **III**  **IV** | 3 (3)  42 (45)  47 (51)  1 (1) | 0 (0)  14 (50)  13 (46)  1 (4) | 1 (8)  4 (33)  7 (58)  0 (0) | 0 (0)  5 (71)  2 (29)  0 (0) | 0 (0)  10 (36)  18 (64)  0 (0) | 2 (11)  9 (50)  7 (39)  0 (0) | -  -  -  - | 0.321 |
| **Preoperative blood results, median (IQR)**  **WBC (x10^3^/µl)**  **Hemoglobin (g/dl)**  **CRP (mg/l)**  **Lipase (U/l)**  **Creatinine (mg/dl)**  **Albumin (g/l)**  **Bilirubin (mg/dl)**  **gGT (U/l)**  **Quick (%)** | 8.1 (4.6)  12.1 (2.4)  6 (17)  36 (53)  0.8 (0.3)  35.4 (11.1)  0.6 (0.6)  87 (194)  93 (21) | 8.9 (6.6)  11.7 (3.4)  9 (18)  52 (71)  0.7 (0.4)  33.7 (10.1)  0.9 (1.7)  146 (269)  92 (14) | 6.8 (5.8)  12.0 (2.0)  7 (12)  51 (48)  0.8 (0.6)  33.9 (7.0)  0.9 (3.4)  123 (358)  84 (22) | 8.2 (8.5)  12.2 (2.6)  3 (3)  38 (250)  0.8 (1.3)  38.3 (12.0)  0.6 (0.4)  36 (77)  96 (23) | 8.0 (4.4)  12.3 (1.9)  6 (21)  39 (92)  0.8 (0.4)  37.1 (12.0)  0.4 (0.3)  84 (330)  93 (22) | 7.9 (4.5)  12.2 (3.6)  5 (13)  22 (23)  0.8 (0.3)  37.9 (13.3)  0.6 (0.3)  50 (129)  95 (21) | -  -  -  -  -  -  -  -  - | 0.550  0.633  0.448  0.246  0.526  0.686  **< 0.001**  0.053  0.826 |
| **Preoperative tumor marker, median (IQR)**  **CEA (ng/ml)**  **CA19-9 (U/ml)** | 3.1 (3.9)  9 (63) | 4.4 (5.6)  403 (4211) | 3.1 (3.6)  24 (131) | 1.4 (1.8)  4 (8) | 3.0 (3.8)  8 (34) | -  1 (3) | -  - | 0.068  **< 0.001** |

* missing data
