# Supplementary material for: Discrimination of pancreato-biliary cancer and pancreatitis patients by non-invasive liquid biopsy
Source: Mol Cancer. 2024 Feb 2;23:28. doi: 10.1186/s12943-024-01943-x (PMC10836044; doi:10.1186/s12943-024-01943-x)
Supplement: Supplementary file 3 — Additional File 3 Overview of 115 individual patients included in the two phases of the study [file 12943_2024_1943_MOESM3_ESM.docx]

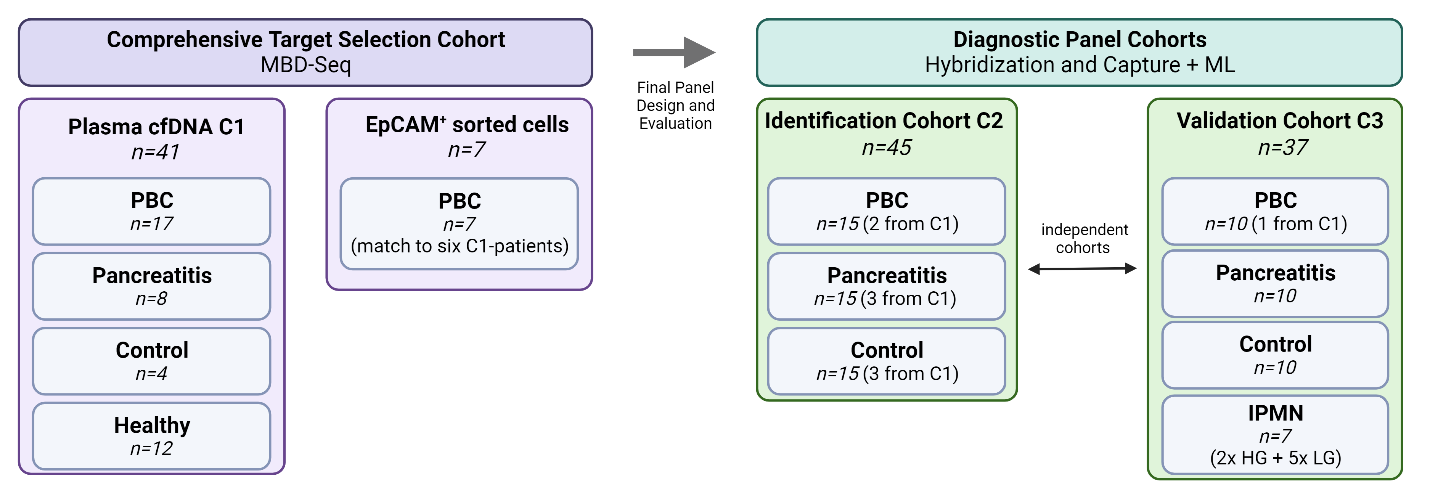


For the comprehensive target selection phase (MBD-Seq), 48 sequencing datasets are presented with cfMBD-Seq of 41 patients (C1) and MBD-Seq of sorted tumor tissue cells (EpCAM+) of 7 patients of which 6 match the corresponding cfMBD-Seq cohort C1. For the diagnostic panel (Hybridization and Capture approach with machine learning=ML), 82 sequencing datasets are included in the study. 45 individuals were included in the identification cohort C2 and 37 independent patients in the validation cohort C3. Overlaps with the cfMBD-Seq cohort C1 are indicated if cfDNA of one individual was used in both phases of the study. HG: high grade; LG: low grade.
