# Supplementary material for: Discrimination of pancreato-biliary cancer and pancreatitis patients by non-invasive liquid biopsy
Source: Mol Cancer. 2024 Feb 2;23:28. doi: 10.1186/s12943-024-01943-x (PMC10836044; doi:10.1186/s12943-024-01943-x)
Supplement: Supplementary file 4 — Additional File 4 Tumor characteristics of patients with PBC (n = 40) [file 12943_2024_1943_MOESM4_ESM.docx]

|  | **All PBC** | **PDAC** | **Non-PDAC** | **p** |
| --- | --- | --- | --- | --- |
| **Neoadjuvant therapy, n (%)** | 5 (13) | 5 (18) | 0 (0) | 0.298 |
| **UICC tumor stage (n=34)*, n (%)**  **0**  **I**  **II**  **III**  **IV** | 1 (3)  3 (9)  14 (41)  13 (38)  3 (9) | 1 (4)  2 (9)  12 (52)  6 (26)  2 (9) | 0 (0)  1 (9)  2 (18)  7 (64)  1 (9) | 0.113 |
| **(y)pT (n=35)*, n (%)**  **Tis**  **1**  **2**  **3**  **4** | 1 (3)  3 (9)  13 (37)  16 (46)  2 (6) | 1 (4)  2 (9)  11 (48)  7 (33)  2 (9) | 0 (0)  1 (8)  2 (17)  9 (75)  0 (0) | 0.161 |
| **(y)pN+ (n=34)*, n (%)** | 19 (56) | 15 (68) | 4 (33) | 1.000 |
| **L+ (n=32)*, n (%)** | 9 (28) | 8 (40) | 1 (8) | 0.103 |
| **V+ (n=32)*, n (%)** | 9 (28) | 8 (40) | 1 (8) | 0.103 |
| **Pn+ (n=34)*, n (%)** | 25 (74) | 18 (82) | 7 (58) | 0.224 |
| **Resection margin (n = 31)*, n (%)**  **R0**  **R1** | 29 (94)  2 (6) | 19 (100)  0 (0) | 10 (83)  2 (17) | 0.142 |
| **Grading (n=33)*, n (%)**  **I**  **II**  **III** | 1 (3)  8 (24)  24 (73) | 1 (5)  4 (19)  16 (76) | 0 (0)  4 (33)  8 (67) | 0.468 |

L = invasion into lymphatic vessels, V = invasion into vein, Pn = perineural invasion.

* missing data
