# Supplementary material for: Discrimination of pancreato-biliary cancer and pancreatitis patients by non-invasive liquid biopsy
Source: Mol Cancer. 2024 Feb 2;23:28. doi: 10.1186/s12943-024-01943-x (PMC10836044; doi:10.1186/s12943-024-01943-x)
Supplement: Supplementary file 5 — Additional File 5: Tumor characteristics of patients with PBC according to subtype (I – III) [file 12943_2024_1943_MOESM5_ESM.docx]

|  | **All PBC** | **Subtype I** | **Subtype II** | **Subtype III** | **p** |
| --- | --- | --- | --- | --- | --- |
| **N (%)** | 40 | 19 (48) | 9 (23) | 12 (30) | - |
| **Age (years), median (IQR)** | 72 (14) | 72 (19) | 68 (13) | 74 (17) | 0.501 |
| **Gender, n (%)**  **Female**  **Male** | 16 (40)  24 (60) | 10 (53)  9 (47) | 3 (33)  6 (67) | 3 (25)  79(75) | 0.314 |
| **BMI (kg/m^2^), median (IQR)** | 24.8 (6.6) | 22.6 (6.8) | 27.4 (7.4) | 26.0 (7.1) | 0.248 |
| **ASA, n (%)**  **I**  **II**  **III**  **IV** | 1 (3)  18 (45)  20 (50)  1 (3) | 0 (0)  8 (42)  11 (58)  0 (0) | 0 (0)  6 (67)  2 (22)  1 (11) | 1 (8)  4 (33)  7 (58)  0 (0) | 0.120 |
| **Neoadjuvant therapy, n (%)** | 5 (13) | 4 (21) | 1 (11) | 0 (0) | 0.228 |
| **UICC tumor stage (n=34), n (%)**  **0**  **I**  **II**  **III**  **IV** | 1 (3)  3 (9)  14 (41)  13 (38)  3 (9) | 1 (7)  1 (7)  6 (40)  5 (33)  2 (14) | 0 (0)  1 (13)  6 (75)  1 (13)  0 (0) | 0 (0)  1 (9)  2 (18)  7 (64)  1 (9) | 0.209 |
| **pT (n=35), n (%)**  **Tis**  **1**  **2**  **3**  **4** | 1 (3)  3 (9)  13 (37)  16 (46)  2 (6) | 1 (7)  1 (7)  6 (40)  6 (40)  1 (7) | 0 (0)  1 (13)  5 (63)  1 (13)  1 (13) | 0 (0)  1 (8)  2 (17)  9 (75)  0 (0) | 0.152 |
| **pN+ (n=34)*, n (%)** | 19 (56) | 10 (67) | 5 (71) | 4 (33) | 0.068 |
| **L+ (n=32)*, n (%)** | 9 (28) | 6 (46) | 2 (29) | 1 (8) | 0.130 |
| **V+ (n=32)*, n (%)** | 9 (28) | 6 (46) | 2 (29) | 1 (8) | 0.130 |
| **Pn+ (n=34)*, n (%)** | 25 (74) | 11 (79) | 7 (88) | 7 (58) | 0.369 |
| **Resection margin (n = 31)*, n (%)**  **R0**  **R1** | 29 (94)  2 (6) | 12 (100)  0 (0) | 7 (100)  0 (0) | 10 (83)  2 (17) | 0.329 |
| **Grading (n=33)*, n (%)**  **I**  **II**  **III** | 1 (3)  8 (24)  24 (73) | 1 (7)  2 (14)  11 (79) | 0 (0)  2 (19)  5 (71) | 0 (0)  4 (33)  8 (67) | 0.447 |

L = invasion into lymphatic vessels, V = invasion into vein, Pn = perineural invasion.

* missing data
