# Supplementary material for: Discrimination of pancreato-biliary cancer and pancreatitis patients by non-invasive liquid biopsy
Source: Mol Cancer. 2024 Feb 2;23:28. doi: 10.1186/s12943-024-01943-x (PMC10836044; doi:10.1186/s12943-024-01943-x)
Supplement: Supplementary file 6 — Additional File 6: Characteristics of pancreatitis patients [file 12943_2024_1943_MOESM6_ESM.docx]

|  | **All patients**  **(n = 30)** | **Patients with premalignant lesions**  **(n = 10)** | **Patients without premalignant lesions**  **(n = 20)** | **p** |
| --- | --- | --- | --- | --- |
| **Age (years), median (IQR)** | 60 (14) | 61 (19) | 58 (13) | 0.217 |
| **Gender, n (%)**  **Female**  **Male** | 10 (33)  20 (67) | 4 (40)  6 (60) | 6 (30)  14 (70) | 0.690 |
| **Localization, n (%)**  **Pancreatic head**  **Pancreatic corpus**  **Pancreatic tail**  **Entire pancreas** | 20 (67)  3 (10)  5 (17)  2 (7) | 8 (80)  1 (10)  1 (10)  0 (0) | 12 (60)  2 (10)  4 (20)  2 (10) | 0.768 |
| **Recurrent pain, n (%)** | 23 (77) | 5 (50) | 18 (90) | **0.026** |
| **Alcohol, n (%)** | 14 (47) | 1 (10) | 13 (65) | **0.007** |
| **Smoking, n (%)** | 14 (47) | 3 (30) | 11 (55) | 0.260 |
| **Preoperative CA19-9 (U/l), median (IQR)** | 8.1 (34.6) | 3.5 (33.2) | 12.3 (35.5) | 0.460 |
| **Preoperative imaging, n (%)**  **CT**  **MRI** | 30 (100)  15 (50) | 10 (100)  7 (70) | 20 (100)  8 (40) | -  0.245 |
| **Kind of pancreatitis, n (%)**  **Acute**  **Chronic** | 3 (10)  27 (90) | 1 (10)  9 (90) | 2 (10)  18 (90) | 1.000 |
| **Premalignant lesions, n (%)** | 10 (33) | 10 (100) | 0 (0) | - |
| **Atlanta classification*, n (%)**  **Moderate**  **Severe** | 1 (33)  2 (67) | 1 (100)  0 (0) | 0 (0)  2 (100) | 0.259 |
| **Cambridge classification**, n (%)**  **0**  **1**  **2**  **3**  **4** | 5 (19)  0 (0)  3 (11)  3 (11)  16 (59) | 5 (56)  0 (0)  1 (11)  1 (11)  2 (22) | 0 (0)  0 (0)  2 (11)  2 (11)  14 (78) | **0.002** |

CT = computed tomography; MRI = magnetic resonance imaging; * only patients with acute pancreatitis (n = 3); ** only patients with chronic pancreatitis (n = 27).
