# Supplementary material for: Discrimination of pancreato-biliary cancer and pancreatitis patients by non-invasive liquid biopsy
Source: Mol Cancer. 2024 Feb 2;23:28. doi: 10.1186/s12943-024-01943-x (PMC10836044; doi:10.1186/s12943-024-01943-x)
Supplement: Supplementary file 7 — Additional File 7: Characteristics of patients with Intraductal Papillary Mucinous Neoplasia (IPMN) [file 12943_2024_1943_MOESM7_ESM.docx]

|  | **IPMN (n = 7)** |
| --- | --- |
| **Age (years), median (IQR)** | 73 (14) |
| **Gender, n (%)**  **Female**  **Male** | 3 (43)  4 (57) |
| **Localization, n (%)**  **Pancreatic head**  **Pancreatic tail** | 5 (71)  2 (29) |
| **Preoperative CA19-9 (U/l), median (IQR)** | 4.3 (8.7) |
| **Preoperative imaging, n (%)**  **MRI + CT**  **MRI + Endosonography + CT**  **MRI + Endosonography**  **Endosonography + CT** | 3 (43)  2 (29)  1 (14)  1 (14) |
| **Diameter of cystic lesion (mm), median (IQR)** | 22 (23) |
| **Indication status according to [1], n (%)**  **Absolute**  **Relative** | 5 (71)  2 (29) |
| **Postoperative histology, n (%)**  **Main duct IPMN**  **Mixed type IPMN**  **Branch duct IPMN** | 4 (57)  2 (29)  1 (14) |
| **Dysplasia in histology, n (%)**  **High-grade dysplasia**  **Low-grade dysplasia** | 2 (29)  5 (71) |

MRI = magnetic resonance imaging; CT = computed tomography. [1] European Study Group on Cystic Tumours of the Pancreas. European evidence-based guidelines on pancreatic cystic neoplasms. Gut. 2018 May;67(5):789-804. doi: 10.1136/gutjnl-2018-316027. Epub 2018 Mar 24. PMID: 29574408; PMCID: PMC5890653.
