# Supplementary material for: Discrimination of pancreato-biliary cancer and pancreatitis patients by non-invasive liquid biopsy
Source: Mol Cancer. 2024 Feb 2;23:28. doi: 10.1186/s12943-024-01943-x (PMC10836044; doi:10.1186/s12943-024-01943-x)
Supplement: Supplementary file 8 — Additional File 8: Visualization of cfMBD-Seq data [file 12943_2024_1943_MOESM8_ESM.docx]

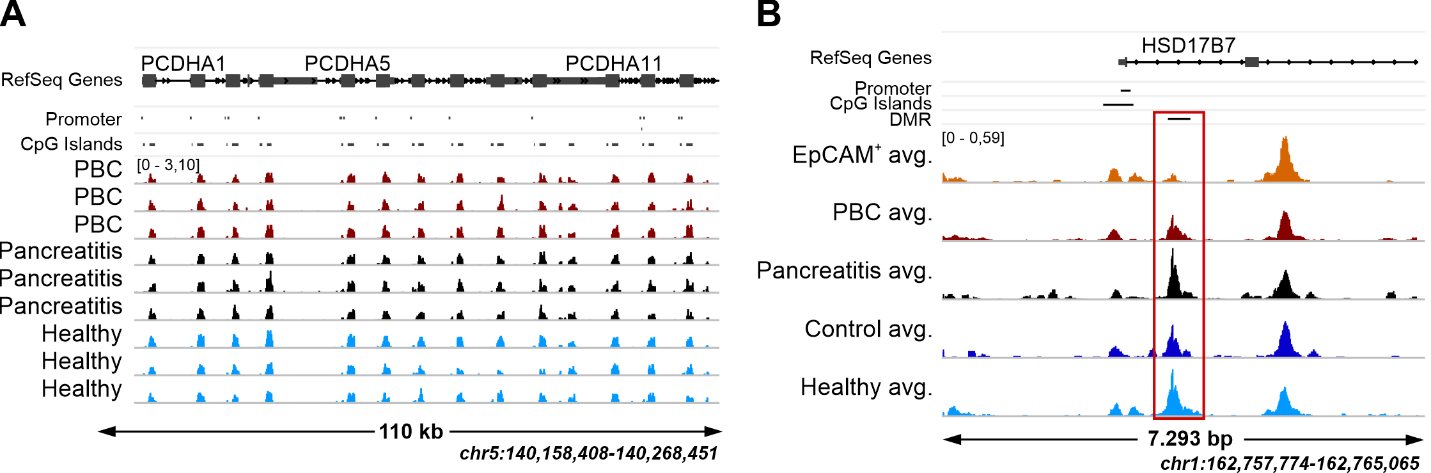


Genomic regions in IGV with annotated RefSeq genes, positions of promoter-like structures annotated by ENCODE and annotated CpG islands are shown. A: Reference region in protocadherin alpha (PCDHA) gene cluster with enrichment of sequencing reads in several CpG islands for different conditions. Red: PBC; black: pancreatitis; light blue: healthy control. B: Visualization of one DMR uniquely hypomethylated in PBCs, averages of for 7 PBC patients’ EpCAM^+^sorted cells, 17 PBCs, 8 pancreatitis, 4 controls, and 12 healthy controls are shown. Identified DMR is indicated. Orange: EpCAM^+^ cells of PDAC patients; red: PBC; black: pancreatitis; dark blue: control; light blue: healthy control.
