# Supplementary material for: Discrimination of pancreato-biliary cancer and pancreatitis patients by non-invasive liquid biopsy
Source: Mol Cancer. 2024 Feb 2;23:28. doi: 10.1186/s12943-024-01943-x (PMC10836044; doi:10.1186/s12943-024-01943-x)
Supplement: Supplementary file 9 — Additional File 9: Analysis of cfMBD-Seq data for detection of DMRs by methylaction [file 12943_2024_1943_MOESM9_ESM.docx]

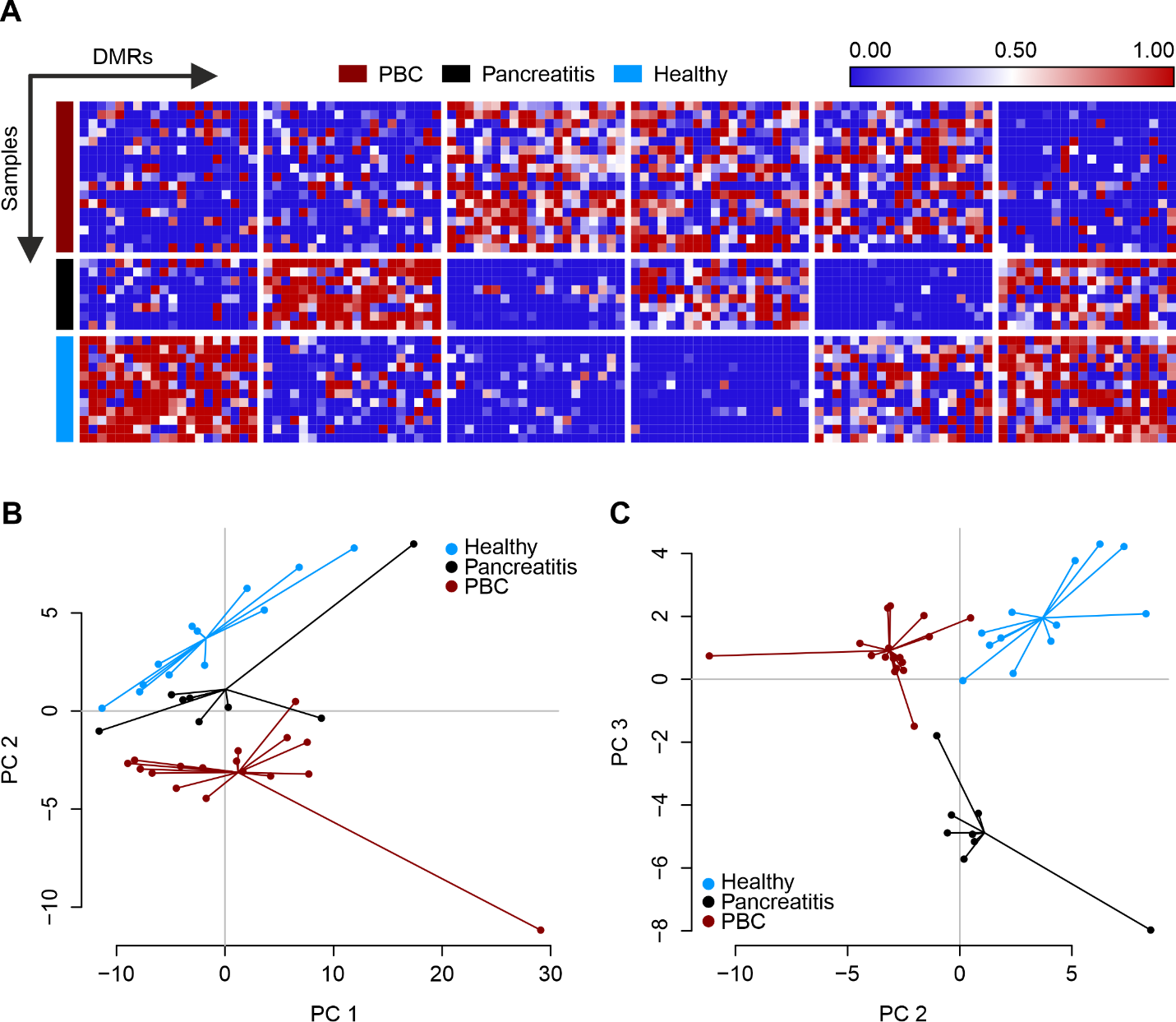


A: Heatmap visualization for the top 120 unqiue DMRs are shown (sum of read counts per patient was scaled to 1 and then scaled with a robust Z-score per DMR). Samples were split by patient groups and DMRs were split by same methylation patterns. B and C: PCA for top 120 DMRs identified by methylaction. PBC shown in red, pancreatitis in black, and healthy in light blue. Variances explained: PC1 = 68.5%, PC2 = 15.9%, PC3 = 8.6%. Lines connect sample points to the centroid of their respective group.
