# Supplementary material for: Discrimination of pancreato-biliary cancer and pancreatitis patients by non-invasive liquid biopsy
Source: Mol Cancer. 2024 Feb 2;23:28. doi: 10.1186/s12943-024-01943-x (PMC10836044; doi:10.1186/s12943-024-01943-x)
Supplement: Supplementary file 10 — Additional File 10: PCA of cfMBD-Seq data with DMRs identified by methylaction [file 12943_2024_1943_MOESM10_ESM.docx]

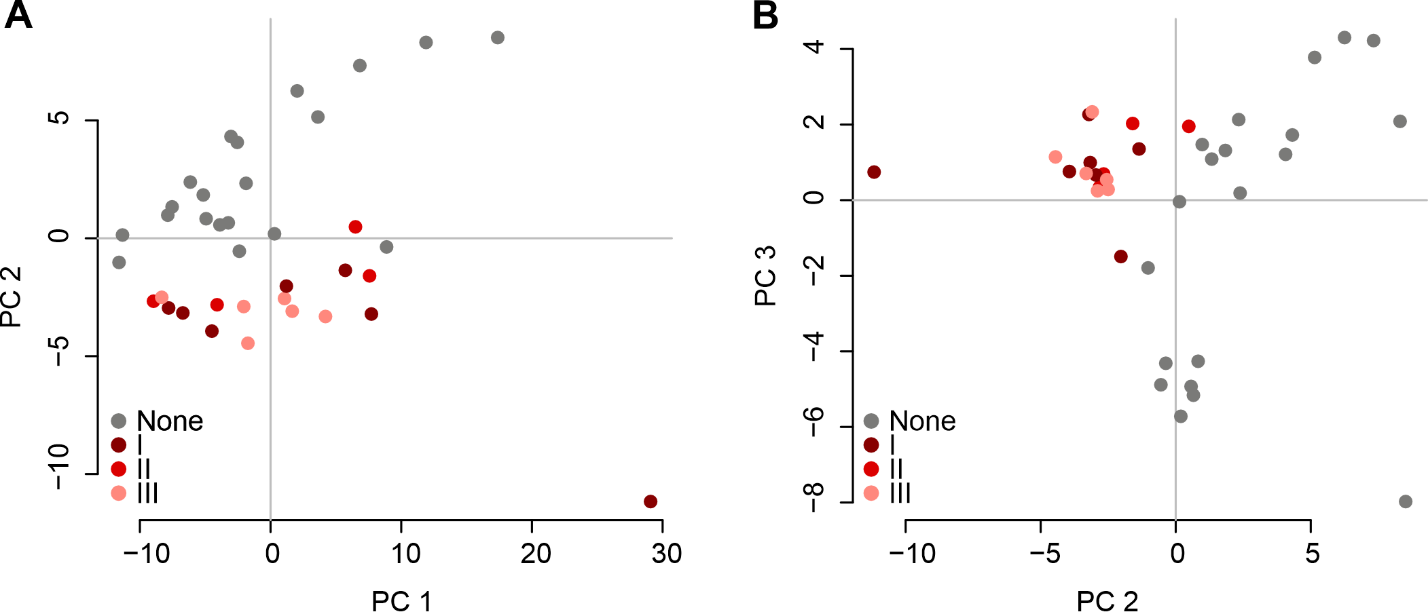


PBC group subgroups are shown for cohort C1. Gray: control samples (none), red: cancer in the head of the pancreas (PDAC) or papillary cancer (I), light red: cancer in the corpus or tail of the pancreas (PDAC; II), rose: non-PDAC (III). A: PC1 vs. PC2. B: PC2 vs. PC3. Variances explained: PC1 = 68.5%, PC2 = 15.9%, PC3 = 8.6%.
