# Supplementary material for: Discrimination of pancreato-biliary cancer and pancreatitis patients by non-invasive liquid biopsy
Source: Mol Cancer. 2024 Feb 2;23:28. doi: 10.1186/s12943-024-01943-x (PMC10836044; doi:10.1186/s12943-024-01943-x)
Supplement: Supplementary file 11 — Additional File 11: 233 DMRs covered by the hybridization and capture panel [file 12943_2024_1943_MOESM11_ESM.docx]

| **DMR number** | **Source** | **Chromosome** | **Start** | **End** |
| --- | --- | --- | --- | --- |
| 1 | revision_of_literature_biomarkers | 3 | 37034841 | 37092337 |
| 2 | revision_of_literature_biomarkers | 10 | 131265454 | 131565783 |
| 3 | revision_of_literature_biomarkers | 11 | 1074875 | 1104417 |
| 4 | revision_of_literature_biomarkers | 11 | 100999808 | 101030001 |
| 5 | revision_of_literature_biomarkers | 11 | 17741110 | 17743678 |
| 6 | revision_of_literature_biomarkers | 11 | 2904448 | 2906995 |
| 7 | revision_of_literature_biomarkers | 15 | 83924655 | 83953468 |
| 8 | revision_of_literature_biomarkers | 3 | 140770743 | 140867453 |
| 9 | revision_of_literature_biomarkers | 4 | 134070470 | 134112732 |
| 10 | revision_of_literature_biomarkers | 8 | 41119476 | 41166990 |
| 11 | revision_of_literature_biomarkers | 9 | 21967751 | 21994490 |
| 12 | revision_of_literature_biomarkers | 9 | 90113885 | 90323549 |
| 13 | revision_of_literature_biomarkers | 3 | 50367217 | 50378367 |
| 14 | revision_of_literature_biomarkers | 3 | 25469754 | 25639422 |
| 15 | revision_of_literature_biomarkers | 7 | 130131899 | 130146138 |
| 16 | revision_of_literature_biomarkers | 1 | 25226002 | 25291475 |
| 17 | revision_of_literature_biomarkers | 13 | 48870649 | 48877797 |
| 18 | revision_of_literature_biomarkers | 21 | 28208606 | 28217728 |
| 19 | revision_of_literature_biomarkers | 3 | 187386694 | 187388201 |
| 20 | revision_of_literature_biomarkers | 4 | 41222091 | 41258744 |
| 21 | revision_of_literature_biomarkers | 4 | 81952119 | 81978685 |
| 22 | revision_of_literature_biomarkers | 4 | 154701742 | 154710228 |
| 23 | revision_of_literature_biomarkers | 5 | 9546312 | 9550409 |
| 24 | revision_of_literature_biomarkers | 5 | 112073556 | 112181936 |
| 25 | revision_of_literature_biomarkers | 5 | 151040657 | 151066615 |
| 26 | revision_of_literature_biomarkers | 7 | 27135713 | 27139877 |
| 27 | revision_of_literature_biomarkers | 7 | 93514709 | 93520303 |
| 28 | revision_of_literature_biomarkers | 7 | 98246597 | 98259181 |
| 29 | revision_of_literature_biomarkers | 8 | 57353513 | 57359282 |
| 30 | revision_of_literature_biomarkers | 12 | 4382902 | 4414522 |
| 31 | revision_of_literature_biomarkers | 6 | 152128814 | 152424408 |
| 32 | MEDIPS_and_methylaction_cfDNA_DMRs | 7 | 27127400 | 27127900 |
| 33 | MEDIPS_and_methylaction_cfDNA_DMRs | 6 | 33032346 | 33048555 |
| 34 | MEDIPS_and_methylaction_cfDNA_DMRs | 1 | 621096 | 622034 |
| 35 | MEDIPS_and_methylaction_cfDNA_DMRs | 1 | 29563028 | 29653325 |
| 36 | MEDIPS_and_methylaction_cfDNA_DMRs | 1 | 36787632 | 36788849 |
| 37 | MEDIPS_and_methylaction_cfDNA_DMRs | 10 | 97071530 | 97145062 |
| 38 | MEDIPS_and_methylaction_cfDNA_DMRs | 10 | 132896569 | 133058707 |
| 39 | MEDIPS_and_methylaction_cfDNA_DMRs | 10 | 134421419 | 134596984 |
| 40 | MEDIPS_and_methylaction_cfDNA_DMRs | 11 | 3379157 | 3400452 |
| 41 | MEDIPS_and_methylaction_cfDNA_DMRs | 11 | 28131713 | 28147409 |
| 42 | MEDIPS_and_methylaction_cfDNA_DMRs | 11 | 49973943 | 49974971 |
| 43 | MEDIPS_and_methylaction_cfDNA_DMRs | 11 | 61640998 | 61659006 |
| 44 | MEDIPS_and_methylaction_cfDNA_DMRs | 12 | 6881681 | 6887621 |
| 45 | MEDIPS_and_methylaction_cfDNA_DMRs | 12 | 50344524 | 50352664 |
| 46 | MEDIPS_and_methylaction_cfDNA_DMRs | 12 | 109877342 | 109886176 |
| 47 | MEDIPS_and_methylaction_cfDNA_DMRs | 12 | 133200348 | 133235485 |
| 48 | MEDIPS_and_methylaction_cfDNA_DMRs | 14 | 29236278 | 29239483 |
| 49 | MEDIPS_and_methylaction_cfDNA_DMRs | 14 | 100172334 | 100193638 |
| 50 | MEDIPS_and_methylaction_cfDNA_DMRs | 14 | 101536248 | 101539273 |
| 51 | MEDIPS_and_methylaction_cfDNA_DMRs | 14 | 106938455 | 106951529 |
| 52 | MEDIPS_and_methylaction_cfDNA_DMRs | 15 | 21040701 | 21071977 |
| 53 | MEDIPS_and_methylaction_cfDNA_DMRs | 15 | 31231151 | 31283807 |
| 54 | MEDIPS_and_methylaction_cfDNA_DMRs | 15 | 92937140 | 93011958 |
| 55 | MEDIPS_and_methylaction_cfDNA_DMRs | 15 | 98503933 | 98517068 |
| 56 | MEDIPS_and_methylaction_cfDNA_DMRs | 16 | 1583658 | 1605581 |
| 57 | MEDIPS_and_methylaction_cfDNA_DMRs | 16 | 15248707 | 15248859 |
| 58 | MEDIPS_and_methylaction_cfDNA_DMRs | 16 | 86612115 | 86615304 |
| 59 | MEDIPS_and_methylaction_cfDNA_DMRs | 16 | 87636493 | 87731761 |
| 60 | MEDIPS_and_methylaction_cfDNA_DMRs | 16 | 89642176 | 89663654 |
| 61 | MEDIPS_and_methylaction_cfDNA_DMRs | 17 | 26800664 | 26821123 |
| 62 | MEDIPS_and_methylaction_cfDNA_DMRs | 17 | 44107282 | 44119280 |
| 63 | MEDIPS_and_methylaction_cfDNA_DMRs | 17 | 71353509 | 71433961 |
| 64 | MEDIPS_and_methylaction_cfDNA_DMRs | 17 | 76108999 | 76118830 |
| 65 | MEDIPS_and_methylaction_cfDNA_DMRs | 17 | 78923236 | 78940173 |
| 66 | MEDIPS_and_methylaction_cfDNA_DMRs | 17 | 80332201 | 80333370 |
| 67 | MEDIPS_and_methylaction_cfDNA_DMRs | 17 | 80709940 | 80901062 |
| 68 | MEDIPS_and_methylaction_cfDNA_DMRs | 18 | 34851241 | 34854641 |
| 69 | MEDIPS_and_methylaction_cfDNA_DMRs | 18 | 77160326 | 77289323 |
| 70 | MEDIPS_and_methylaction_cfDNA_DMRs | 18 | 77160326 | 77289323 |
| 71 | MEDIPS_and_methylaction_cfDNA_DMRs | 18 | 77905807 | 77936315 |
| 72 | MEDIPS_and_methylaction_cfDNA_DMRs | 19 | 2100987 | 2129162 |
| 73 | MEDIPS_and_methylaction_cfDNA_DMRs | 19 | 58907457 | 58908446 |
| 74 | MEDIPS_and_methylaction_cfDNA_DMRs | 2 | 113587337 | 113593203 |
| 75 | MEDIPS_and_methylaction_cfDNA_DMRs | 2 | 127413511 | 127454251 |
| 76 | MEDIPS_and_methylaction_cfDNA_DMRs | 2 | 132480064 | 132524977 |
| 77 | MEDIPS_and_methylaction_cfDNA_DMRs | 2 | 232260335 | 232265875 |
| 78 | MEDIPS_and_methylaction_cfDNA_DMRs | 2 | 237994084 | 238004527 |
| 79 | MEDIPS_and_methylaction_cfDNA_DMRs | 2 | 240882432 | 240882511 |
| 80 | MEDIPS_and_methylaction_cfDNA_DMRs | 20 | 62189439 | 62199107 |
| 81 | MEDIPS_and_methylaction_cfDNA_DMRs | 22 | 47169824 | 47571342 |
| 82 | MEDIPS_and_methylaction_cfDNA_DMRs | 4 | 4269429 | 4281267 |
| 83 | MEDIPS_and_methylaction_cfDNA_DMRs | 4 | 6641818 | 6644470 |
| 84 | MEDIPS_and_methylaction_cfDNA_DMRs | 5 | 473334 | 524549 |
| 85 | MEDIPS_and_methylaction_cfDNA_DMRs | 5 | 1461542 | 1467440 |
| 86 | MEDIPS_and_methylaction_cfDNA_DMRs | 5 | 123972610 | 123980354 |
| 87 | MEDIPS_and_methylaction_cfDNA_DMRs | 5 | 123982372 | 124084500 |
| 88 | MEDIPS_and_methylaction_cfDNA_DMRs | 5 | 132157833 | 132162002 |
| 89 | MEDIPS_and_methylaction_cfDNA_DMRs | 5 | 157212751 | 157286183 |
| 90 | MEDIPS_and_methylaction_cfDNA_DMRs | 5 | 169532917 | 169536729 |
| 91 | MEDIPS_and_methylaction_cfDNA_DMRs | 6 | 397056 | 402637 |
| 92 | MEDIPS_and_methylaction_cfDNA_DMRs | 6 | 32008932 | 32077151 |
| 93 | MEDIPS_and_methylaction_cfDNA_DMRs | 6 | 33540323 | 33543225 |
| 94 | MEDIPS_and_methylaction_cfDNA_DMRs | 6 | 168185219 | 168197539 |
| 95 | MEDIPS_and_methylaction_cfDNA_DMRs | 7 | 618893 | 641221 |
| 96 | MEDIPS_and_methylaction_cfDNA_DMRs | 7 | 618893 | 641221 |
| 97 | MEDIPS_and_methylaction_cfDNA_DMRs | 7 | 937537 | 966852 |
| 98 | MEDIPS_and_methylaction_cfDNA_DMRs | 7 | 937537 | 994306 |
| 99 | MEDIPS_and_methylaction_cfDNA_DMRs | 7 | 1473995 | 1482120 |
| 100 | MEDIPS_and_methylaction_cfDNA_DMRs | 7 | 4721930 | 4811074 |
| 101 | MEDIPS_and_methylaction_cfDNA_DMRs | 7 | 5160941 | 5172198 |
| 102 | MEDIPS_and_methylaction_cfDNA_DMRs | 7 | 6448747 | 6457422 |
| 103 | MEDIPS_and_methylaction_cfDNA_DMRs | 7 | 102128377 | 102135315 |
| 104 | MEDIPS_and_methylaction_cfDNA_DMRs | 7 | 102191679 | 102202757 |
| 105 | MEDIPS_and_methylaction_cfDNA_DMRs | 7 | 123670970 | 123673523 |
| 106 | MEDIPS_and_methylaction_cfDNA_DMRs | 8 | 118811602 | 119124058 |
| 107 | MEDIPS_and_methylaction_cfDNA_DMRs | 8 | 144391556 | 144407648 |
| 108 | MEDIPS_and_methylaction_cfDNA_DMRs | 9 | 92254698 | 92334674 |
| 109 | MEDIPS_and_methylaction_cfDNA_DMRs | 9 | 124964856 | 124984019 |
| 110 | MEDIPS_and_methylaction_cfDNA_DMRs | 9 | 139715781 | 139735639 |
| 111 | MEDIPS_and_methylaction-Top120_cfDNA_DMRs | 11 | 72396114 | 72424288 |
| 112 | MEDIPS_and_methylaction-Top120_cfDNA_DMRs | 12 | 6802957 | 6810009 |
| 113 | MEDIPS_and_methylaction-Top120_cfDNA_DMRs | 12 | 51674822 | 51717938 |
| 114 | MEDIPS_and_methylaction-Top120_cfDNA_DMRs | 14 | 103388993 | 103397179 |
| 115 | MEDIPS_and_methylaction-Top120_cfDNA_DMRs | 16 | 593277 | 593366 |
| 116 | MEDIPS_and_methylaction-Top120_cfDNA_DMRs | 17 | 21825370 | 21826499 |
| 117 | MEDIPS_and_methylaction-Top120_cfDNA_DMRs | 17 | 72931897 | 72946087 |
| 118 | MEDIPS_and_methylaction-Top120_cfDNA_DMRs | 18 | 77287119 | 77289323 |
| 119 | MEDIPS_and_methylaction-Top120_cfDNA_DMRs | 2 | 65283495 | 65301312 |
| 120 | MEDIPS_and_methylaction-Top120_cfDNA_DMRs | 22 | 19553653 | 19554362 |
| 121 | MEDIPS_and_methylaction-Top120_cfDNA_DMRs | 3 | 152880029 | 152886263 |
| 122 | MEDIPS_and_methylaction-Top120_cfDNA_DMRs | 5 | 176758563 | 176778885 |
| 123 | MEDIPS_and_methylaction-Top120_cfDNA_DMRs | 7 | 12726911 | 12730558 |
| 124 | MEDIPS_and_methylaction-Top120_cfDNA_DMRs | 7 | 63081468 | 63081547 |
| 125 | MEDIPS_and_methylaction-Top120_cfDNA_DMRs | 7 | 102227551 | 102234484 |
| 126 | MEDIPS_and_methylaction-Top120_cfDNA_DMRs | 8 | 145754563 | 145911194 |
| 127 | methylaction-Top120_cfDNA_DMRs | 1 | 2938046 | 2939467 |
| 128 | methylaction-Top120_cfDNA_DMRs | 1 | 157061835 | 157069600 |
| 129 | methylaction-Top120_cfDNA_DMRs | 1 | 162760496 | 162782608 |
| 130 | methylaction-Top120_cfDNA_DMRs | 1 | 220960039 | 220987741 |
| 131 | methylaction-Top120_cfDNA_DMRs | 10 | 12237961 | 12258491 |
| 132 | methylaction-Top120_cfDNA_DMRs | 10 | 50822350 | 50901939 |
| 133 | methylaction-Top120_cfDNA_DMRs | 10 | 73199584 | 73407219 |
| 134 | methylaction-Top120_cfDNA_DMRs | 10 | 127262940 | 127267014 |
| 135 | methylaction-Top120_cfDNA_DMRs | 10 | 131265454 | 131565783 |
| 136 | methylaction-Top120_cfDNA_DMRs | 11 | 70049269 | 70053508 |
| 137 | methylaction-Top120_cfDNA_DMRs | 12 | 131438452 | 131626008 |
| 138 | methylaction-Top120_cfDNA_DMRs | 13 | 111293757 | 111358480 |
| 139 | methylaction-Top120_cfDNA_DMRs | 14 | 77972340 | 78083110 |
| 140 | methylaction-Top120_cfDNA_DMRs | 14 | 95648276 | 95786245 |
| 141 | methylaction-Top120_cfDNA_DMRs | 14 | 106135888 | 106139144 |
| 142 | methylaction-Top120_cfDNA_DMRs | 15 | 29362861 | 29394962 |
| 143 | methylaction-Top120_cfDNA_DMRs | 15 | 42134711 | 42138580 |
| 144 | methylaction-Top120_cfDNA_DMRs | 15 | 43307854 | 43398286 |
| 145 | methylaction-Top120_cfDNA_DMRs | 15 | 82722988 | 82731586 |
| 146 | methylaction-Top120_cfDNA_DMRs | 16 | 1583658 | 1605581 |
| 147 | methylaction-Top120_cfDNA_DMRs | 16 | 21807951 | 21830495 |
| 148 | methylaction-Top120_cfDNA_DMRs | 16 | 29086163 | 29128038 |
| 149 | methylaction-Top120_cfDNA_DMRs | 16 | 34980923 | 34990995 |
| 150 | methylaction-Top120_cfDNA_DMRs | 16 | 64980683 | 65038944 |
| 151 | methylaction-Top120_cfDNA_DMRs | 16 | 88493879 | 88507165 |
| 152 | methylaction-Top120_cfDNA_DMRs | 17 | 2965963 | 2966901 |
| 153 | methylaction-Top120_cfDNA_DMRs | 17 | 29861901 | 29861989 |
| 154 | methylaction-Top120_cfDNA_DMRs | 17 | 80900031 | 80964864 |
| 155 | methylaction-Top120_cfDNA_DMRs | 18 | 14337422 | 14342523 |
| 156 | methylaction-Top120_cfDNA_DMRs | 18 | 74690789 | 74844774 |
| 157 | methylaction-Top120_cfDNA_DMRs | 19 | 926037 | 972803 |
| 158 | methylaction-Top120_cfDNA_DMRs | 19 | 3094408 | 3121454 |
| 159 | methylaction-Top120_cfDNA_DMRs | 19 | 3976054 | 3985461 |
| 160 | methylaction-Top120_cfDNA_DMRs | 19 | 15510746 | 15529833 |
| 161 | methylaction-Top120_cfDNA_DMRs | 19 | 17905919 | 17924385 |
| 162 | methylaction-Top120_cfDNA_DMRs | 19 | 47931279 | 47969676 |
| 163 | methylaction-Top120_cfDNA_DMRs | 19 | 55219601 | 55224887 |
| 164 | methylaction-Top120_cfDNA_DMRs | 2 | 64319786 | 64325364 |
| 165 | methylaction-Top120_cfDNA_DMRs | 2 | 97163383 | 97171094 |
| 166 | methylaction-Top120_cfDNA_DMRs | 2 | 132480064 | 132524977 |
| 167 | methylaction-Top120_cfDNA_DMRs | 2 | 239756673 | 239795893 |
| 168 | methylaction-Top120_cfDNA_DMRs | 20 | 30053309 | 30060816 |
| 169 | methylaction-Top120_cfDNA_DMRs | 20 | 61732644 | 61735737 |
| 170 | methylaction-Top120_cfDNA_DMRs | 21 | 43782391 | 43786644 |
| 171 | methylaction-Top120_cfDNA_DMRs | 21 | 44834398 | 44847002 |
| 172 | methylaction-Top120_cfDNA_DMRs | 21 | 46825097 | 46933634 |
| 173 | methylaction-Top120_cfDNA_DMRs | 22 | 19318224 | 19419219 |
| 174 | methylaction-Top120_cfDNA_DMRs | 22 | 39745954 | 39827887 |
| 175 | methylaction-Top120_cfDNA_DMRs | 22 | 39994156 | 40081215 |
| 176 | methylaction-Top120_cfDNA_DMRs | 3 | 27465132 | 27525911 |
| 177 | methylaction-Top120_cfDNA_DMRs | 3 | 195701268 | 195717150 |
| 178 | methylaction-Top120_cfDNA_DMRs | 4 | 154631312 | 154681387 |
| 179 | methylaction-Top120_cfDNA_DMRs | 5 | 9053928 | 9054007 |
| 180 | methylaction-Top120_cfDNA_DMRs | 5 | 167956582 | 167957639 |
| 181 | methylaction-Top120_cfDNA_DMRs | 5 | 174151575 | 174157902 |
| 182 | methylaction-Top120_cfDNA_DMRs | 6 | 3722836 | 3752246 |
| 183 | methylaction-Top120_cfDNA_DMRs | 6 | 91223292 | 91297020 |
| 184 | methylaction-Top120_cfDNA_DMRs | 7 | 1581871 | 1596066 |
| 185 | methylaction-Top120_cfDNA_DMRs | 7 | 74508347 | 74565623 |
| 186 | methylaction-Top120_cfDNA_DMRs | 7 | 80371854 | 80551675 |
| 187 | methylaction-Top120_cfDNA_DMRs | 7 | 155592736 | 155601766 |
| 188 | methylaction-Top120_cfDNA_DMRs | 8 | 8175258 | 8239257 |
| 189 | methylaction-Top120_cfDNA_DMRs | 9 | 13105703 | 13279563 |
| 190 | methylaction-Top120_cfDNA_DMRs | 9 | 102584137 | 102596341 |
| 191 | methylaction-Top120_cfDNA_DMRs | 9 | 133320094 | 133376661 |
| 192 | methylaction-Top120_and_SICER_cfDNA_DMRs | 7 | 1473995 | 1482120 |
| 193 | SICER_cfDNA_DMRs | 1 | 162466964 | 162499419 |
| 194 | SICER_cfDNA_DMRs | 10 | 55568452 | 56424050 |
| 195 | SICER_cfDNA_DMRs | 10 | 88516396 | 88684945 |
| 196 | SICER_cfDNA_DMRs | 11 | 69954459 | 70035652 |
| 197 | SICER_cfDNA_DMRs | 12 | 57482677 | 57489259 |
| 198 | SICER_cfDNA_DMRs | 12 | 120123595 | 120242282 |
| 199 | SICER_cfDNA_DMRs | 16 | 70841287 | 70900232 |
| 200 | SICER_cfDNA_DMRs | 17 | 17584787 | 17714765 |
| 201 | SICER_cfDNA_DMRs | 2 | 10580508 | 10588453 |
| 202 | SICER_cfDNA_DMRs | 21 | 45432206 | 45480110 |
| 203 | SICER_cfDNA_DMRs | 4 | 183811244 | 183838630 |
| 204 | SICER_cfDNA_DMRs | 4 | 186990309 | 187006252 |
| 205 | SICER_cfDNA_DMRs | 5 | 131630145 | 131679899 |
| 206 | SICER_cfDNA_DMRs | 7 | 99578385 | 99581860 |
| 207 | SICER_cfDNA_DMRs | 8 | 1806921 | 1852264 |
| 208 | SICER_cfDNA_DMRs | 8 | 47752508 | 47765991 |
| 209 | SICER_cfDNA_DMRs | 8 | 47752508 | 47765991 |
| 210 | SICER_cfDNA_DMRs | 8 | 55047781 | 55061074 |
| 211 | TCGA_tissue | 6 | 41514164 | 41570122 |
| 212 | TCGA_tissue | 1 | 50883223 | 50887304 |
| 213 | TCGA_tissue | 1 | 111214310 | 111217655 |
| 214 | TCGA_tissue | 1 | 200008658 | 200146550 |
| 215 | TCGA_tissue | 10 | 129535538 | 129539450 |
| 216 | TCGA_tissue | 10 | 132890655 | 133109984 |
| 217 | TCGA_tissue | 12 | 2785167 | 2800323 |
| 218 | TCGA_tissue | 13 | 110801310 | 110959496 |
| 219 | TCGA_tissue | 15 | 83316521 | 83361572 |
| 220 | TCGA_tissue | 15 | 83776324 | 83806111 |
| 221 | TCGA_tissue | 16 | 1883984 | 1922179 |
| 222 | TCGA_tissue | 18 | 5392388 | 5543986 |
| 223 | TCGA_tissue | 19 | 30863328 | 31048965 |
| 224 | TCGA_tissue | 2 | 25042039 | 25142055 |
| 225 | TCGA_tissue | 2 | 133014539 | 133014653 |
| 226 | TCGA_tissue | 3 | 147103835 | 147110217 |
| 227 | TCGA_tissue | 4 | 5564146 | 5710294 |
| 228 | TCGA_tissue | 4 | 9783258 | 9785633 |
| 229 | TCGA_tissue | 5 | 178405330 | 178422124 |
| 230 | TCGA_tissue | 7 | 49813257 | 49952138 |
| 231 | TCGA_tissue | 7 | 101460882 | 101901513 |
| 232 | TCGA_tissue | 7 | 157367028 | 157367114 |
| 233 | TCGA_tissue | 8 | 141541264 | 141645646 |

Source for identification, as well as chromosomal position, are indicated (GRCh37).
