# Supplementary figures and images for: Discrimination of pancreato-biliary cancer and pancreatitis patients by non-invasive liquid biopsy
Source: Mol Cancer. 2024 Feb 2;23:28. doi: 10.1186/s12943-024-01943-x (PMC10836044; doi:10.1186/s12943-024-01943-x)

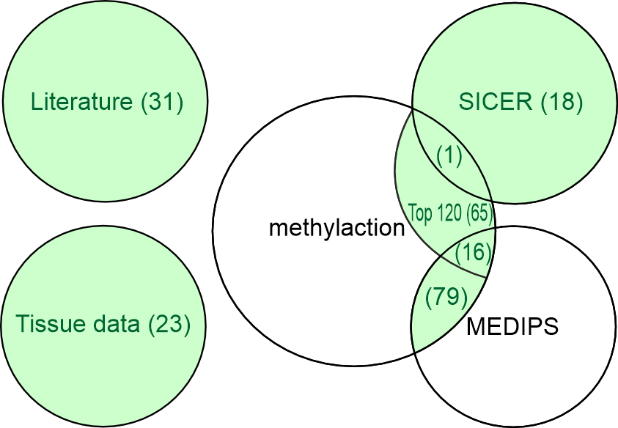


Sources for identification are indicated.

Supplement: Supplementary file 12 — Additional File 12: Venn diagram for the 233 DMRs covered by the hybridization and capture sequencing panel [file 12943_2024_1943_MOESM12_ESM.docx]

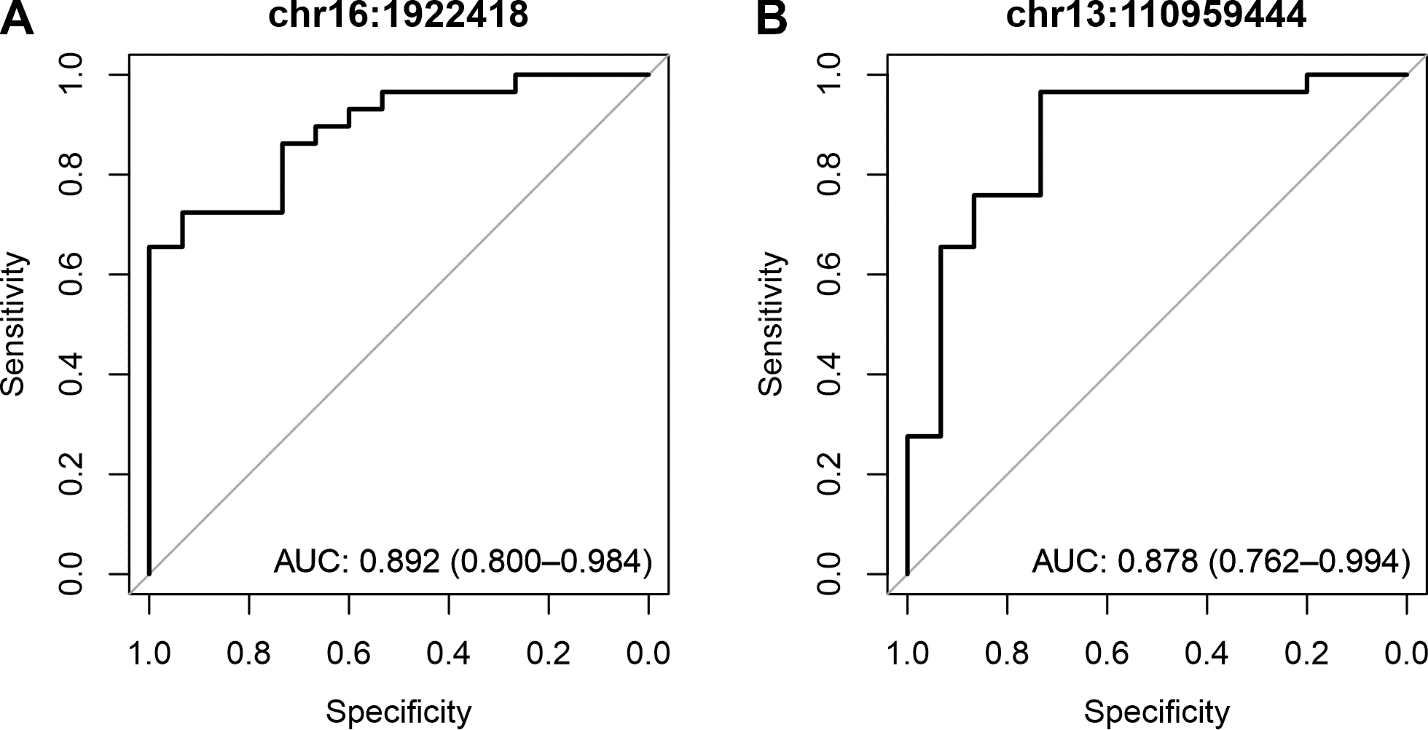


ROC curves for the discrimination of PBCs from pancreatitis (15 PBCs, 15 pancreatitis, and 14 controls).

Supplement: Supplementary file 20 — Additional File 20: Methylation markers on different chromosomes identified with the hybridization and capture sequencing [file 12943_2024_1943_MOESM20_ESM.docx]

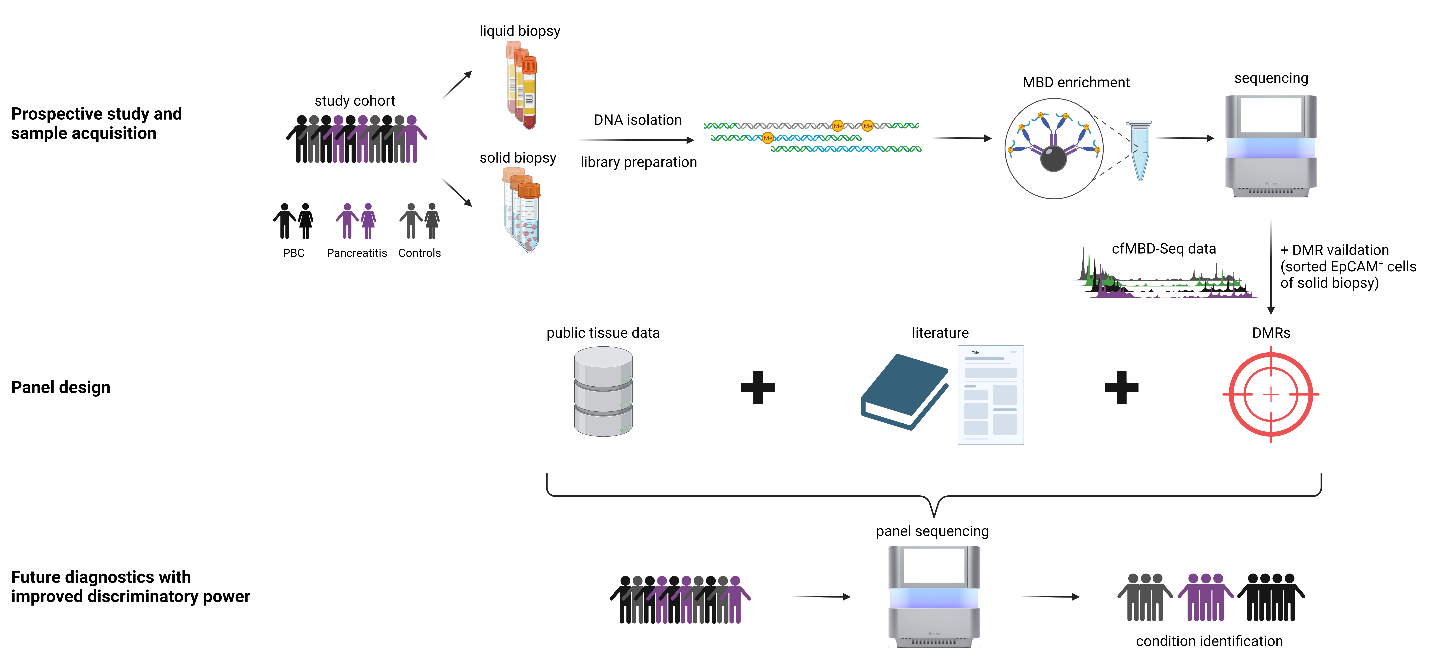

Supplement: Supplementary file 24 — Additional File 24: Graphical abstract [file 12943_2024_1943_MOESM24_ESM.docx]
