# Supplementary material for: Discrimination of pancreato-biliary cancer and pancreatitis patients by non-invasive liquid biopsy
Source: Mol Cancer. 2024 Feb 2;23:28. doi: 10.1186/s12943-024-01943-x (PMC10836044; doi:10.1186/s12943-024-01943-x)
Supplement: Supplementary file 13 — Additional File 13: Validation of cohort characteristics of cohort C2 [file 12943_2024_1943_MOESM13_ESM.docx]

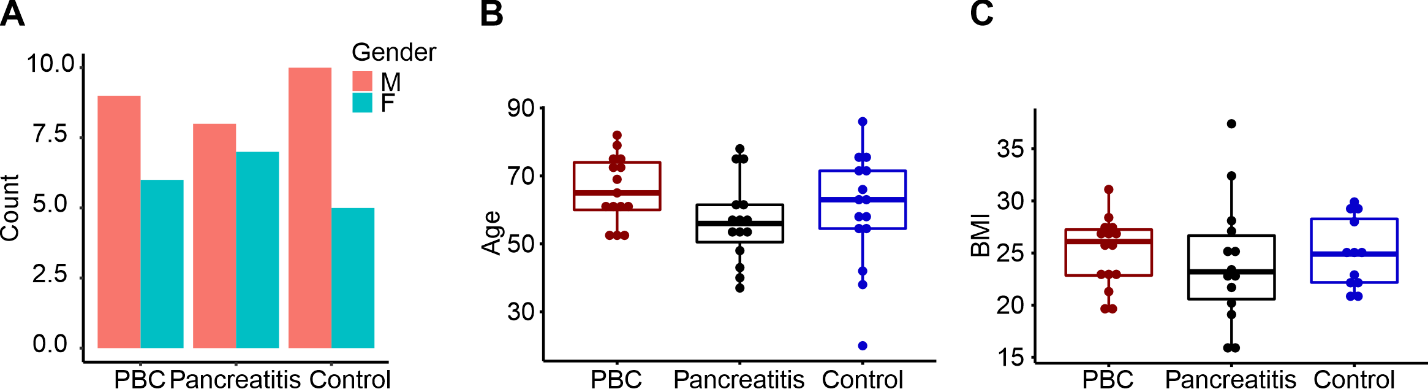


Gender, age, and BMI are not significantly different between the three groups PBC, pancreatitis, and controls in the identitification cohort C2 and are thereby excluded as confounding factors.
