# Supplementary material for: Discrimination of pancreato-biliary cancer and pancreatitis patients by non-invasive liquid biopsy
Source: Mol Cancer. 2024 Feb 2;23:28. doi: 10.1186/s12943-024-01943-x (PMC10836044; doi:10.1186/s12943-024-01943-x)
Supplement: Supplementary file 14 — Additional File 14: 50 DMCs identified as putative biomarkers by the hybridization and capture panel [file 12943_2024_1943_MOESM14_ESM.docx]

| **DMC rank** | **Source** | **Chromosome** | **Position** |
| --- | --- | --- | --- |
| 1 | methylaction-Top120_cfDNA_DMRs | chr7 | 80811653 |
| 2 | SICER_cfDNA_DMRs | chr8 | 47339485 |
| 3 | methylaction-Top120_cfDNA_DMRs | chr7 | 80811652 |
| 4 | MEDIPS_and_methylaction-Top120_cfDNA_DMRs | chr12 | 6818325 |
| 5 | revision_of_literature_biomarkers | chr15 | 83953342 |
| 6 | MEDIPS_and_methylaction-Top120_cfDNA_DMRs | chr12 | 6818323 |
| 7 | TCGA_tissue | chr13 | 110959444 |
| 8 | SICER_cfDNA_DMRs | chr8 | 47127217 |
| 9 | SICER_cfDNA_DMRs | chr8 | 47339484 |
| 10 | SICER_cfDNA_DMRs | chr8 | 47339478 |
| 11 | TCGA_tissue | chr16 | 1922418 |
| 12 | MEDIPS_and_methylaction_cfDNA_DMRs | chr1 | 36788188 |
| 13 | MEDIPS_and_methylaction_cfDNA_DMRs | chr7 | 630582 |
| 14 | TCGA_tissue | chr15 | 83776697 |
| 15 | TCGA_tissue | chr1 | 50886941 |
| 16 | TCGA_tissue | chr7 | 49813448 |
| 17 | TCGA_tissue | chr1 | 111217174 |
| 18 | revision_of_literature_biomarkers | chr5 | 112073538 |
| 19 | methylaction-Top120_cfDNA_DMRs | chr14 | 106130663 |
| 20 | revision_of_literature_biomarkers | chr5 | 112073427 |
| 21 | TCGA_tissue | chr13 | 110959452 |
| 22 | revision_of_literature_biomarkers | chr5 | 112073434 |
| 23 | TCGA_tissue | chr16 | 1922383 |
| 24 | MEDIPS_and_methylaction-Top120_cfDNA_DMRs | chr8 | 145912882 |
| 25 | MEDIPS_and_methylaction_cfDNA_DMRs | chr7 | 630597 |
| 26 | TCGA_tissue | chr7 | 49813425 |
| 27 | TCGA_tissue | chr15 | 83316716 |
| 28 | MEDIPS_and_methylaction_cfDNA_DMRs | chr18 | 77190043 |
| 29 | TCGA_tissue | chr1 | 111216974 |
| 30 | revision_of_literature_biomarkers | chr5 | 112073407 |
| 31 | TCGA_tissue | chr1 | 111216960 |
| 32 | MEDIPS_and_methylaction_cfDNA_DMRs | chr1 | 29587252 |
| 33 | MEDIPS_and_methylaction_cfDNA_DMRs | chr1 | 29587251 |
| 34 | MEDIPS_and_methylaction_cfDNA_DMRs | chr11 | 49913449 |
| 35 | MEDIPS_and_methylaction-Top120_cfDNA_DMRs | chr12 | 6818355 |
| 36 | MEDIPS_and_methylaction_cfDNA_DMRs | chr1 | 36788223 |
| 37 | revision_of_literature_biomarkers | chr5 | 112073398 |
| 38 | revision_of_literature_biomarkers | chr5 | 112073571 |
| 39 | MEDIPS_and_methylaction_cfDNA_DMRs | chr1 | 36787996 |
| 40 | MEDIPS_and_methylaction_cfDNA_DMRs | chr1 | 36788216 |
| 41 | revision_of_literature_biomarkers | chr5 | 112073439 |
| 42 | MEDIPS_and_methylaction_cfDNA_DMRs | chr20 | 62198807 |
| 43 | MEDIPS_and_methylaction_cfDNA_DMRs | chr7 | 630568 |
| 44 | methylaction-Top120_cfDNA_DMRs | chr5 | 173942318 |
| 45 | revision_of_literature_biomarkers | chr15 | 83953320 |
| 46 | MEDIPS_and_methylaction_cfDNA_DMRs | chr7 | 630581 |
| 47 | revision_of_literature_biomarkers | chr5 | 112073490 |
| 48 | MEDIPS_and_methylaction_cfDNA_DMRs | chr1 | 36788221 |
| 49 | MEDIPS_and_methylaction_cfDNA_DMRs | chr7 | 630569 |
| 50 | MEDIPS_and_methylaction_cfDNA_DMRs | chr1 | 36788060 |

Source for identification, as well as chromosomal position, are indicated (GRCh37).
