# Supplementary material for: Discrimination of pancreato-biliary cancer and pancreatitis patients by non-invasive liquid biopsy
Source: Mol Cancer. 2024 Feb 2;23:28. doi: 10.1186/s12943-024-01943-x (PMC10836044; doi:10.1186/s12943-024-01943-x)
Supplement: Supplementary file 15 — Additional File 15: Schematic view of the machine learning approach for unbiased performance evaluation [file 12943_2024_1943_MOESM15_ESM.docx]

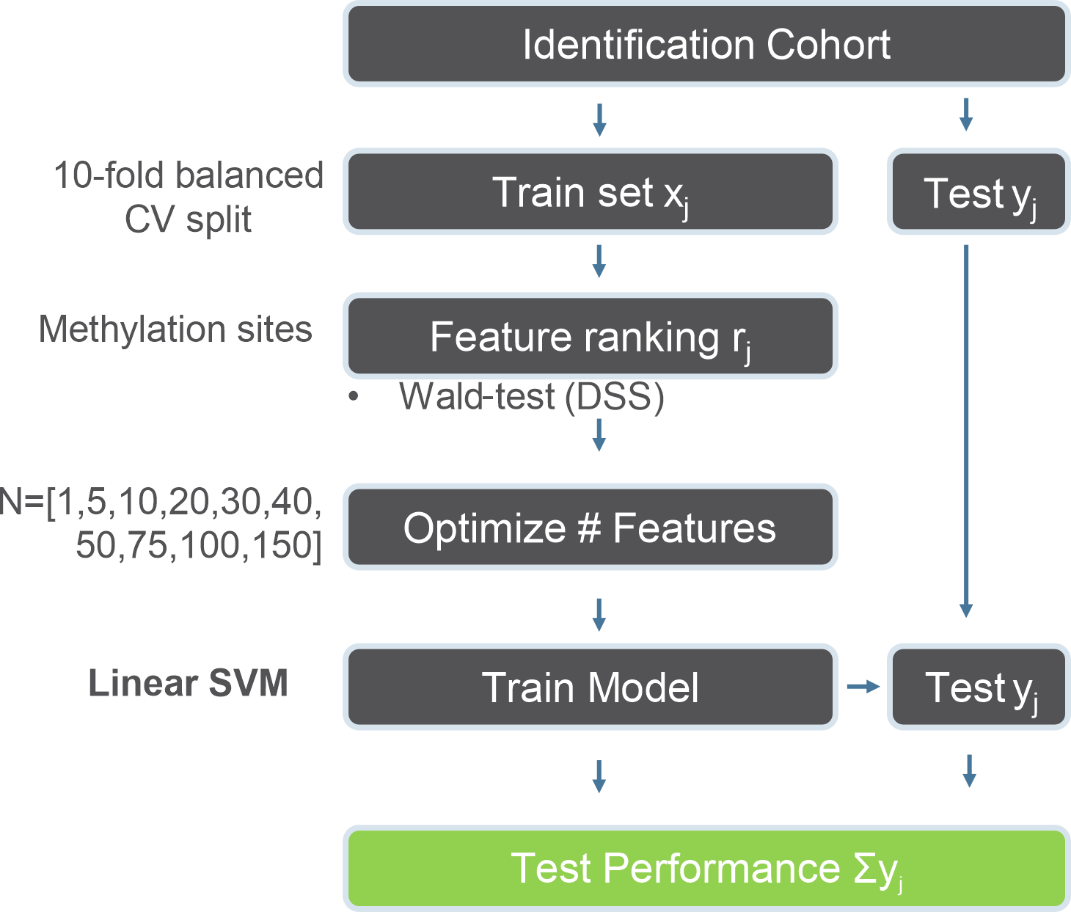


Samples from 15 PBC, 15 pancreatitis, and 15 clinical control patients were used as identification cohort C2 followed by a validation cohort C3 using samples from 10 PBC, 7 IPMNs (2 high grade and 5 low grade), 10 pancreatitis, and 10 clinical control patients.
