# Supplementary material for: Discrimination of pancreato-biliary cancer and pancreatitis patients by non-invasive liquid biopsy
Source: Mol Cancer. 2024 Feb 2;23:28. doi: 10.1186/s12943-024-01943-x (PMC10836044; doi:10.1186/s12943-024-01943-x)
Supplement: Supplementary file 17 — Additional File 17: Machine learning approach M1 [file 12943_2024_1943_MOESM17_ESM.docx]

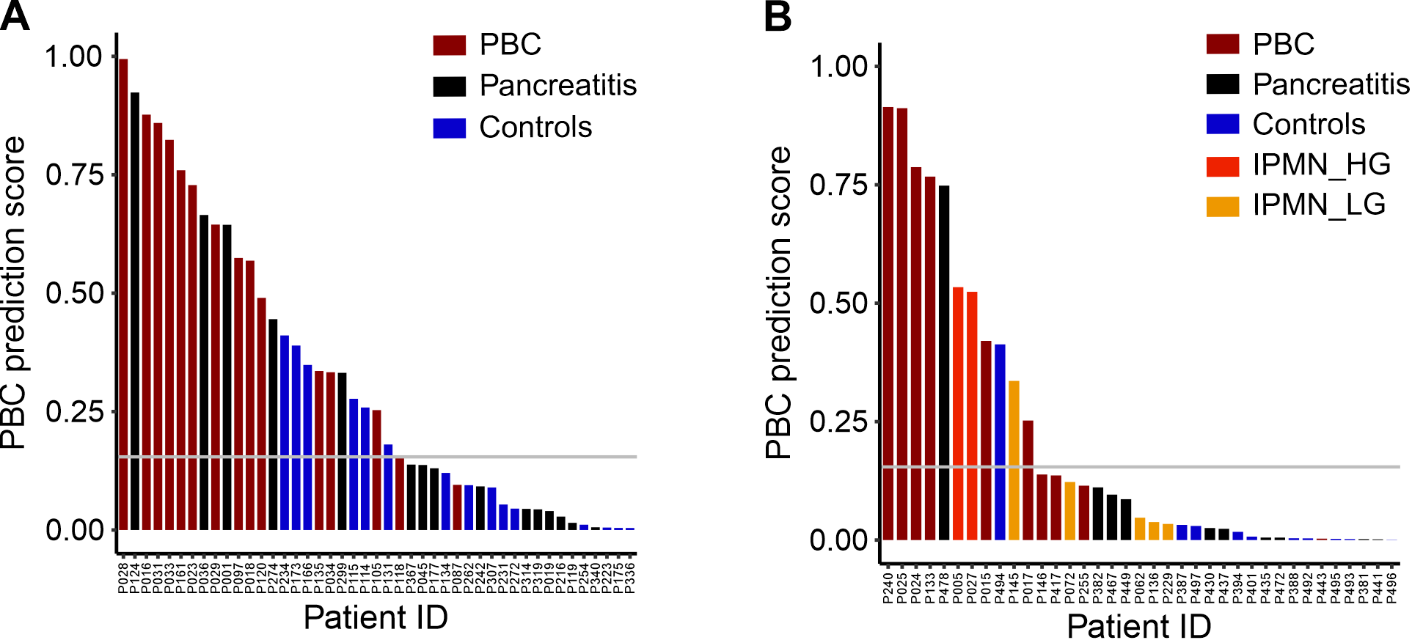


A: PBC prediction scores from the identification cohort C2 shown per patient sorted by decreasing PBC prediction scores. The classification threshold (0.15) to maximize the sum of sensitivity and specificity is indicated by a gray line. B: PBC prediction scores from the validation cohort C3 including low and high grade IPMNs shown per patient sorted by decreasing PBC prediction scores.
