# Supplementary material for: Discrimination of pancreato-biliary cancer and pancreatitis patients by non-invasive liquid biopsy
Source: Mol Cancer. 2024 Feb 2;23:28. doi: 10.1186/s12943-024-01943-x (PMC10836044; doi:10.1186/s12943-024-01943-x)
Supplement: Supplementary file 18 — Additional File 18: Comparison of identification cohort C2 with validation cohort C3 of the machine learning approach [file 12943_2024_1943_MOESM18_ESM.docx]

|  | **All patients**  **(n = 82)** | **Identification cohort C2**  **(n = 45)** | **Validation cohort C3**  **(n = 37)** | **p** |
| --- | --- | --- | --- | --- |
| **Age (years), median (IQR)** | 65 (17) | 61 (19) | 70 (15) | **0.007** |
| **Gender, n (%)**  **Female**  **Male** | 32 (39)  50 (61) | 18 (40)  27 (60) | 14 (38)  23 (63) | 1.000 |
| **BMI (kg/m^2^) (n=72)*, median (IQR)** | 24.5 (6.2) | 24.8 (5.8) | 24.4 (6.3) | 0.952 |
| **ASA (n=72)*, n (%)**  **I**  **II**  **III**  **IV** | 2 (3)  33 (46)  36 (50)  1 (1) | 1 (2)  19 (45)  22 (52)  0 (0) | 1 (3)  14 (47)  14 (47)  1 (3) | 0.807 |
| **Preoperative blood results, median (IQR)**  **WBC (x10^3^/µl)**  **Hemoglobin (g/dl)**  **CRP (mg/l)**  **Lipase (U/l)**  **Creatinine (mg/dl)**  **Albumin (g/l)**  **Bilirubin (mg/dl)**  **gGT (U/l)**  **Quick (%)** | 8.0 (4.2)  12.1 (2.8)  6 (16)  34 (54)  0.8 (0.3)  36.1 (12.0)  0.6 (0.5)  84 (144)  93 (20) | 7.5 (3.8)  12.1 (2.6)  6 (13)  32 (46)  0.7 (0.4)  35.8 (11.6)  0.6 (0.5)  98 (276)  88 (22) | 8.2 (3.8)  12.3 (2.9)  4 (20)  40 (84)  0.8 (0.5)  38.3 (12.0)  0.6 (0.7)  82 (119)  96 (15) | 0.448  0.246  0.892  0.258  0.102  0.523  0.860  0.380  0.082 |
| **Preoperative tumor marker, median (IQR)**  **CEA (ng/ml)**  **CA19-9 (U/ml)** | 3.1 (3.9)  6 (41) | 3.3 (3.6)  10 (41) | 3.1 (6.5)  3 (52) | 0.846  0.313 |
| **Subtypes, n (%)**  **PDAC**  **Non-PDAC**  **IPMN**  **Pancreatitis**  **Controls** | 18 (22)  7 (9)  7 (9)  25 (31)  25 (31) | 9 (20)  6 (13)  0 (0)  15 (33)  15 (33) | 9 (24)  1 (3)  7 (19)  10 (27)  10 (27) | **0.016** |

* missing data.
