# Supplementary material for: Discrimination of pancreato-biliary cancer and pancreatitis patients by non-invasive liquid biopsy
Source: Mol Cancer. 2024 Feb 2;23:28. doi: 10.1186/s12943-024-01943-x (PMC10836044; doi:10.1186/s12943-024-01943-x)
Supplement: Supplementary file 19 — Additional File 19: Beta values of identification cohort C2 and validation cohort C3 [file 12943_2024_1943_MOESM19_ESM.docx]

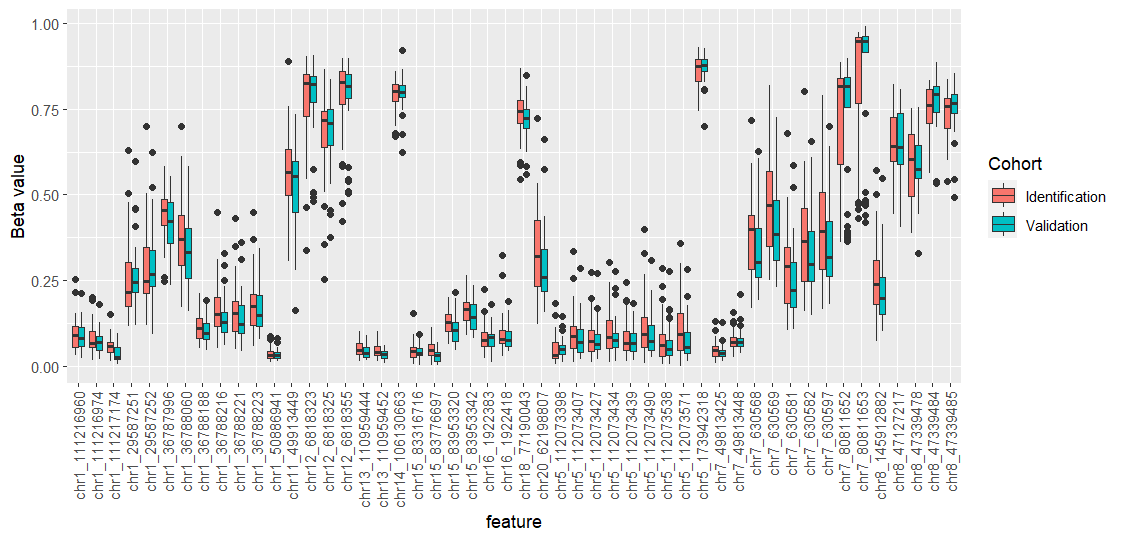


Distribution is shown for all top 50 DMCs (features) identified (chromosome and position) with boxes indicating the interquartile range (IQR) and whiskers extend to the minimum and maximum values within 1.5 times the IQR. Outliers are depicted by points and median by a horizontal line. Identification cohort C2: red boxes (left); validation cohort C3: green boxes (right).
