# Supplementary material for: Discrimination of pancreato-biliary cancer and pancreatitis patients by non-invasive liquid biopsy
Source: Mol Cancer. 2024 Feb 2;23:28. doi: 10.1186/s12943-024-01943-x (PMC10836044; doi:10.1186/s12943-024-01943-x)
Supplement: Supplementary file 21 — Additional File 21: Machine learning approach CA19-9 [file 12943_2024_1943_MOESM21_ESM.docx]

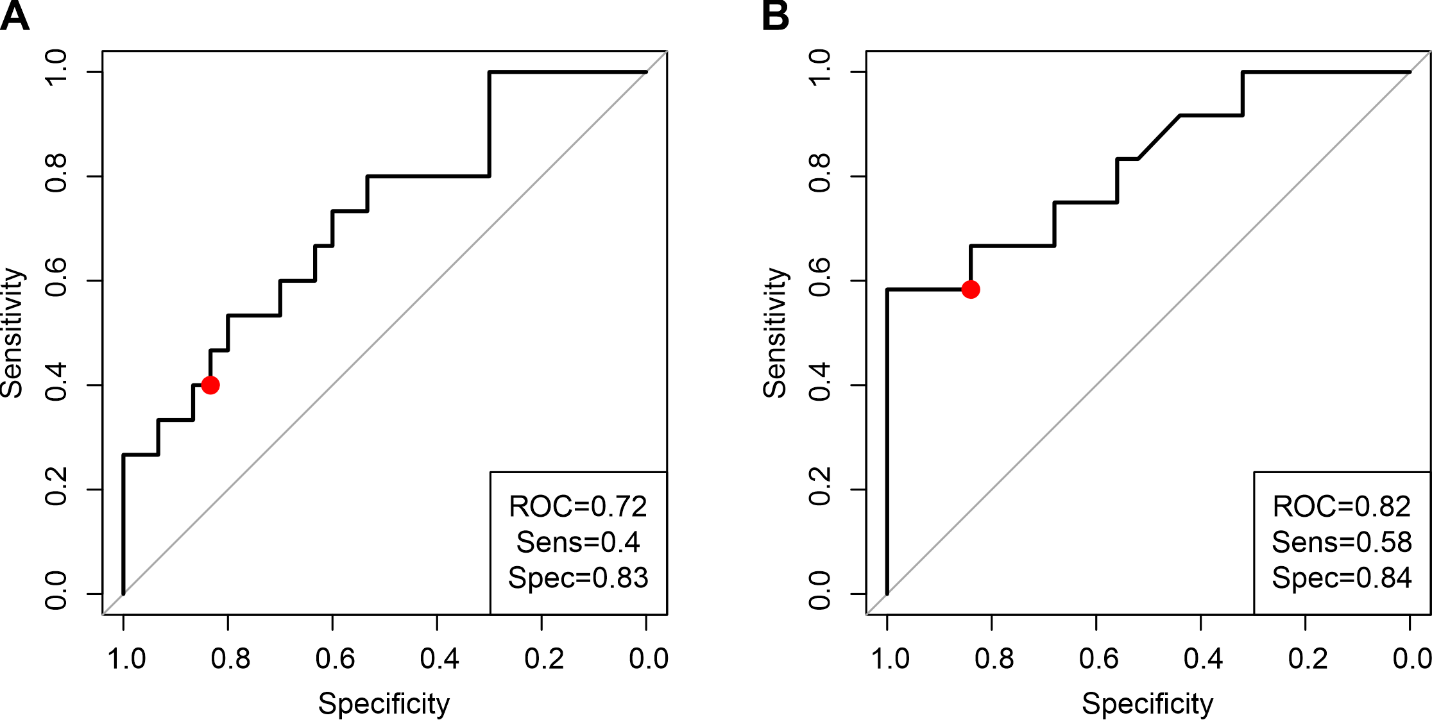


ROC curves for the identification (A) and validation (B) cohort of the machine learning approach considering only CA19-9 values with the cutoff 37 U/ml for the discrimination of positive (PBC, high grade IPMN) from negative (controls, pancreatitis, low grade IPMN) samples. The red dot indicates the determined optimal threshold value that maximizes sensitivity and specificity for classifying PBCs.
